# Long-Term Administration of BTH2 Hypoallergenic Vaccine Candidate Induces Hallmarks of Allergen Immunotherapy in Murine Model of *Blomia tropicalis*-Induced Asthma

**DOI:** 10.3390/biomedicines13112657

**Published:** 2025-10-29

**Authors:** Eduardo Santos da Silva, Antônio Márcio Santana Fernandes, Raphael Chagas Silva, Lorena Miranda de Souza, Jennifer Emily Anunciação Sousa, Carolina Melo Orrico-Ferreira, Neuza Maria Alcântara-Neves, Luis Gustavo Carvalho Pacheco, Carina da Silva Pinheiro

**Affiliations:** Laboratory of Allergology and Acarology, Institute of Health Sciences, Federal University of Bahia, Salvador 40110-902, BA, Brazilcarina.pinheiro@ufba.br (C.d.S.P.)

**Keywords:** allergy, Blo t 5, Blo t 21, hypoallergen, IgG blocking antibodies, mucosal IgA, subcutaneous immunotherapy, treatment

## Abstract

**Background/Objectives**: Allergen-specific immunotherapy remains the only disease-modifying treatment for allergic diseases, and the use of recombinant hypoallergenic derivatives is a promising therapeutic approach. Among these, BTH2 has previously shown efficacy in an acute murine model of allergy induced by *Blomia tropicalis*. The present study aimed to evaluate both the efficacy and safety of BTH2 in a chronic asthma model induced by *B. tropicalis*. **Methods**: A/J male mice (*n* = 6) were sensitized and chronically challenged with *B. tropicalis* extract over four months. One group repeatedly received subcutaneous doses of BTH2 (25 µg) for three months (65 doses). Parameters of allergic airway inflammation, antibody profiles, cytokine levels, and markers of AIT success were evaluated in bronchoalveolar lavage fluid, lung tissue, serum, and splenocyte cultures. **Results**: Repeated BTH2 administration was well tolerated, with no signs of systemic toxicity. BTH2 significantly reduced neutrophilic and eosinophilic airway inflammation, while increasing lymphocytes and regulatory cytokines in the lungs. It suppressed IgE against *B. tropicalis* allergens, while inducing mucosal IgA responses and systemic IgG, which may be linked to the observed blocking antibody activity in BTH2-treated mice. The treatment also led to downregulation of Th2 cytokines and enhanced expression of regulatory and Th1-associated cytokines, especially IL-10, TGF-β and IFN-γ. Correlation matrix analyses indicated that regulatory cytokines were correlated with beneficial antibody responses and reduced inflammation. **Conclusions**: BTH2 shows strong therapeutic and immunomodulatory effects in a chronic asthma model induced by *B. tropicalis*, with a favorable safety profile. These findings support its potential for future clinical trials, including those involving patients with allergic asthma.

## 1. Introduction

Respiratory diseases have long been part of human history and remain a significant public health concern [1,2,3]. Among them, allergic disorders—once considered conditions of high-income countries—are now prevalent worldwide, including across Latin America [4,5,6,7,8,9]. These conditions encompass a range of clinical manifestations, such as allergic asthma, atopic dermatitis, allergic rhinitis, and anaphylaxis. All of them impose a considerable burden on affected individuals and on healthcare systems [3,4,7,10,11,12,13,14]. Asthma may be one of the most concerning illnesses in this list and accounted for an estimated 3340 cases per 100,000 individuals in 2021 [15,16]. Furthermore, the number of cases increased steadily from 1990 to 2021, and projections suggest that the global age-standardized incidence rate will remain elevated from 2022 through 2050 [15,16].

Asthma is an inflammatory airway disease recognized by reversible airflow obstruction and bronchial hyperresponsiveness [3,17,18,19]. Exposure to aeroallergens, for instance, can trigger immune reactions that initiate and sustain the pathophysiology of allergic asthma, potentially contributing to disease chronicity [5,6,18,19]. House dust mites are among the principal allergens implicated in these inflammatory responses, serving as perennial sources that continuously provoke symptoms in individuals with allergic asthma [20,21,22]. In tropical and subtropical regions, *Blomia tropicalis* is considered one of the most clinically relevant species, frequently associated with asthma [23,24,25,26]. However, a recent study showed that sensitization to this mite is not exclusive of a specific climate zone [27].

The treatment for allergic asthma usually centers on pharmacotherapy, including therapeutic regimens with antihistamines, β-blockers, and, most notably, corticosteroids. Although they are still essential to improve patients’ quality of life, these drugs are not curative; the pathological process of asthma may continue to progress despite the suppression of clinical symptoms. Furthermore, their use is associated with significant side effects, particularly in patients requiring long-term treatment [28,29,30].

In contrast to conventional pharmacological approaches, several studies have demonstrated that allergen-specific immunotherapy (AIT) may represent a safer and more effective alternative, being the only disease-modifying treatment available [31,32]. The primary goal of AIT is to induce a tolerogenic immune response, leading to symptomatic improvement by targeting the underlying cause of the disease [33,34]. However, early AIT approaches based on diluted crude extracts are limited by adverse effects, as thoroughly reviewed in the literature [35,36,37,38,39]. Nevertheless, recent developments in the field have advanced AIT significantly. Hypoallergenic derivatives have shown promise in multiple preclinical studies [40,41,42,43,44,45,46] and early-phase clinical trials [47,48,49], offering a safer profile and enabling the design of more specific protein-based therapies. These novel formulations are more precisely tailored to the patient’s allergic profile and may enhance treatment adherence and overall therapeutic efficacy.

Among the various hypoallergenic candidates developed worldwide, our group has successfully engineered some molecules [42,44,50]. BTH2 currently stands as the most advanced candidate within our panel of engineered hypoallergens, backed by the most extensive in vitro and in vivo preclinical evaluation performed to date [43,44]. BTH2 was designed using segments of the major allergens Blo t 5 and Blo t 21, incorporating specific mutations to generate a hybrid molecule with reduced allergenicity, whose schematic representation is detailed in a previous study [44]. This construct achieved several key efficacy parameters, as reported in two different in vivo evaluations of our group [43,44].

However, these experiments used immunization and acute allergy murine models, which assess antibody responses and mimic allergic rhinitis, respectively. A model reflecting asthma chronicity and enabling repeated-dose therapeutic evaluation is still needed. Our group recently developed a chronic model, testing a hypoallergenic hybrid protein as a treatment for *Dermatophagoides pteronyssinus* allergic asthma [51].

In this context, the aim of the present study was to evaluate the preclinical safety and efficacy of BTH2 in a murine model of asthma induced by *Blomia tropicalis*, under a prolonged and repeated-dose treatment protocol. This approach explored whether BTH2 is safe and capable of treating a chronic disease, thereby supporting its potential for future clinical trials in patients with mild to moderate allergic asthma.

## 2. Materials and Methods

### 2.1. Production and Quality Control of Blomia tropicalis Extract and Recombinant Allergens

Our group’s protocol to produce the *Blomia tropicalis* extract (BtE) has been previously described in detail [43,52]. Briefly, mites were isolated from indoor dust samples and cultured in BOD incubators (Solab, Piracicaba, SP, Brazil) until stationary growth phase. Mites were purified by flotation in 5 M NaCl (Dinâmica©, São Paulo, SP, Brazil;), desalted and resuspended in 0.15 mM phosphate-buffered saline (1× PBS; pH 7.4). Lysis was performed under ice bath and protease inhibitor in a blender (Waring Commercial, Torrington, CT, USA). The lysate was centrifuged, and the supernatant was defatted by three successive cycles of incubation with diethyl ether (PA ACS; Hexis Científica, Jundiaí, Brazil) for 30 min, followed by centrifugation at 11,000× *g* for 15 min. Total protein concentration was determined using the Bradford assay [53,54]. To verify the purity and confirm identity of our *B. tropicalis* cultures, we routinely perform species authentication of the biomass using a ribosomal DNA–based multiplex PCR assay, following previously described protocols of our group [55].

The extract was further standardized by quantifying the major allergen Blo t 5 via immunoblotting analysis [56]. A total of 25 µg/per well of BtE and a calibration curve of recombinant Blo t 5 (rBlo t 5; 0.08–5 µg/per well) were resolved by 15% Sodium Dodecyl Sulfate–Polyacrylamide Gel Electrophoresis (SDS-PAGE) and transferred onto a nitrocellulose membrane (BioRad, Hercules, CA, USA, Cat. No. 1620112). The membrane was blocked with 5% non-fat dried milk (Molico, Vevey, Switzerland) prepared in 1× PBS containing 0.3% Tween-20 (Merck, Rahway, NJ, USA; Cat. No. P1379-1L). Anti-mouse IgG against rBlo t 5 (1:3000), obtained from mouse serum characterized in a previous study [57], was employed as the primary antibody to detect both recombinant and native Blo t 5. The membrane was incubated for 1 h at room temperature (RT), followed by incubation with an alkaline phosphatase-conjugated anti-mouse IgG antibody (1:4000, Thermo Fisher Scientific, Waltham, MA, USA; Cat. No. M30108) for 1 h at RT. Immunoreactive bands were visualized using NBT/BCIP substrate solution (Sigma-Aldrich, St. Louis, MO, USA; Cat. No. 11681451001). Densitometric analysis was performed using ImageJ software (version 1.54, NIH, Bethesda, MD, USA) [58] to quantify Blo t 5 levels in BtE. The determined concentration of Blo t 5 was 16.21 ng per µg of total protein. The somatic extract was stored at −20 °C until use.

The recombinant forms of the wild-type allergens (rBlo t 5 and rBlo t 21), as well as the hypoallergenic variant BTH2, were produced and purified as previously described by our group [43,44,57]. Endotoxin (lipopolysaccharide, LPS) content in BtE and recombinant protein samples were quantified using an endpoint chromogenic assay (Thermo Fisher Scientific, Waltham, MA, USA; Cat. No. A39552). When necessary, LPS was removed using the Pierce High-Capacity Endotoxin Removal Resin (Thermo Fisher Scientific, Waltham, MA, USA; Cat. No. 88270). After treatment, the residual LPS levels were reassessed, and all samples used in this study contained endotoxin concentrations below the recommended safety thresholds reported for in vivo studies [59,60]. Specifically, before endotoxin removal, the estimated daily LPS dose per animal was 0.02 EU (50 µL) for BtE and 4.89 EU (150 µL) for BTH2. According to the endotoxin limits in formulations for preclinical research [59,60], only the BTH2 preparation exceeded the recommended threshold. After removal, LPS content in BTH2 formulation corresponded to 0.91 EU per daily dose (150 µL), allowing its use.

### 2.2. Murine Model of Allergic Asthma Induced by Blomia tropicalis

**Figure 1:**
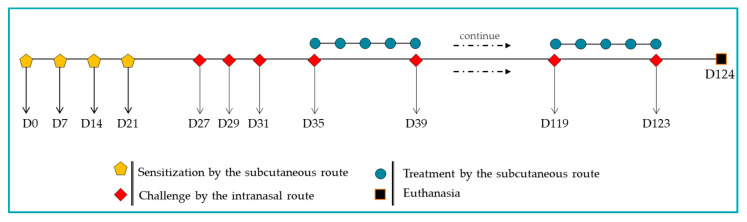
Schematic representation of the murine model of asthma induced by *Blomia tropicalis* and the BTH2 treatment schedule.

The experimental protocol (Figure 1) was adapted from a recent study of our group [51] with specific modifications. A/J male mice (*n* = 6) were sensitized on days 0, 7, 14, and 21 via subcutaneous injections of 100 µg of BtE, adsorbed onto 4 mg/mL of aluminum hydroxide (Alhydrogel^®^ 2%, InvivoGen, San Diego, CA, USA, Cat. No. vac-alu-250) and diluted in endotoxin-free 1× PBS. The negative control group (termed as Control) was given 1× PBS plus Alhydrogel^®^ during sensitization. Nasal challenges were performed on days 27, 29, and 31 by intranasal instillation of 10 µg of BtE per animal. Beginning on day 35, further challenges were delivered twice per week (Mondays and Fridays) and maintained throughout the remainder of the four-month protocol. During the challenge phase, both the positive control (Sham) and BTH2-treated groups received BtE, whereas the Control group was administered with 1× PBS. Therapeutic treatment with 25 µg of BTH2 was administered subcutaneously five times per week (Monday through Friday). These treatments also began on day 35 and were maintained for the remainder of the protocol. No adjuvants were used during treatment. On days when treatments coincided with the challenges, the treatment was performed one hour after the intranasal challenges. Control and Sham groups were treated with 1× PBS injections. On day 124, the animals were euthanized by intraperitoneal injection of a xylazine/ketamine mixture (40 mg/kg/body weight, Syntec, Tamboré, SP, Brazil). At the time of euthanasia, mice presented an average body weight of 17.88 ± 1.13 g.

The experimental design used block randomization without blinding for the treatment phase. All mice were individually identified using tag numbers, and each treatment group was assigned a numerical code for identification. To ensure that the treatment order was randomized each day, a different sequence was generated daily. The specific sequence was created using the RANK.EQ (or ORDEN.EQ in some software versions) function in Microsoft Excel on the tag and group codes before commencing the daily treatments. This procedure ensured that neither the groups nor the individual mice were consistently handled or treated first, thus minimizing the potential for operator bias related to handling order or time of day.

Following tissues’ collection, therapeutic efficacy and safety were assessed based on the quantification of biochemical and inflammatory parameters. All in vivo experiments involving murine models were approved by the Ethics Committee on the Use of Experimental Animals of the Institute of Health Sciences, Federal University of Bahia, Brazil (CEUA protocol no. 8373280920).

### 2.3. Evaluation of Toxicological Effects

Similarly to previous studies, clinical signs and behavioral parameters were monitored in the study [61,62,63]. This included the monitoring of specific signs such as nose rubbing, sneezing frequency, head-down posture, and rearing, as well as an inspection of the application site to assess the animal’s response. Moreover, toxicological effects were further evaluated at the end of the experimental model. Clinical biochemistry analyses included measurements of uric acid, gamma-glutamyl transferase, alanine aminotransferase, and aspartate aminotransferase, providing insight into potential systemic toxicity, particularly hepatic and renal function. All biochemical evaluations were performed by a certified veterinary diagnostic laboratory (Ventilab, Salvador, BA, Brazil), using serum samples. Anatomical assessments involved the collection and weighing of major organs, specifically the lungs, kidneys, spleen, and liver, as indicators of possible inflammatory or toxic alterations [63].

### 2.4. Cell Counts from Bronchoalveolar Lavage Fluid

As previously described [42,43,51,64,65], the tracheas of euthanized mice were cannulated, and the lungs were gently lavaged three times with 0.5 mL of 1× PBS containing 1% bovine serum albumin (1% BSA; Sigma Chemical Co., St. Louis, MO, USA; Cat. No. A9647-500G). The collected bronchoalveolar lavage fluid (BALF) was centrifuged at 2000× *g* for 10 min at 4 °C. Supernatants were stored at −20 °C for subsequent cytokine analysis, while the cell pellet was resuspended in 400 µL of 1× PBS-1% BSA. Total cell counts were performed using a Neubauer chamber following Trypan blue exclusion. For differential cell counts, cytospin slides were prepared using up to 200 µL of the resuspended sample and stained with Romanowsky–Giemsa stain (Panótico Rápido; Laborclin, Pinhais, PR, Brazil; Cat. No. 940069). A minimum of 100 cells per sample were analyzed under blind conditions by two independent researchers and based on standard morphological criteria. The remaining cell suspension was stored at −20 °C for later quantification of eosinophil peroxidase (EPO) activity.

### 2.5. EPO Activity in BALF and Lung Homogenates

EPO activity was assessed using a colorimetric assay as previously described [42,43,51,64]. Following BALF collection, the lungs were perfused via the heart with 5 mL of 1× PBS. The right lung lobe was excised, weighed, and homogenized in cold 1× PBS in the presence of protease inhibitors (1 mM PMSF and 5mM EDTA; Thermo Fisher Scientific, Waltham, MA, USA, Cat. No. 36978 and Promega, Madison, WI, USA, Cat No. H5032, respectively). After centrifugation at 2000× *g* for 10 min at 4 °C, the supernatant was stored at −20 °C for later cytokine quantification. The remaining pellet was treated with red blood cell lysis buffer and further resuspended in 1× PBS containing 0.5% hexadecyltrimethylammonium bromide (HTAB, Sigma-Aldrich, St. Louis, MO, USA; Cat. No. H5882-100G) to a final concentration of 10% (*w*/*v*). On the day of the assay, both BALF cell suspensions and lung debris homogenates were subjected to seven cycles of sonication (15 s each at 25 kHz) interspersed with resting intervals, all performed on ice. Samples were then centrifuged at 2000× *g* for 10 min at 4 °C. Supernatants were transferred to 96-well microplates and incubated with substrate solution containing 1.5 mmol/L o-phenylenediamine (Sigma-Aldrich, St. Louis, MO, USA; Cat. No. P23938-100G) and 6.6 mmol/L H_2_O_2_ (Neon, Suzano, SP, Brazil; Cat. No. 01984) in 0.05 mol/L Tris–HCl buffer (pH 8.0). After a 30 min incubation at room temperature in the dark, the reaction was stopped with 75 µL of 0.2 mol/L citric acid (Êxodo Científica, Sumaré, SP, Brazil; Cat. No. AC09584RA). Absorbance was measured at 492 nm using a Multiskan™ FC Microplate Photometer (Thermo Fisher Scientific, Waltham, MA, USA).

### 2.6. Splenocyte Culture and Stimulation Index

Following blood collection, spleens were harvested and mechanically dissociated to obtain a single-cell suspension. Specifically, spleens were placed in a Petri dish and injected with 2 mL of pyrogen-free saline using a 5 mL syringe. The perfusate was collected and reinjected 7–10 times until the tissue appeared translucent. The resulting cell suspension was transferred to a 50 mL Falcon tube. The remaining tissue was then macerated through a wire mesh in 1 mL of pyrogen-free saline. The cell suspension and macerated tissue were combined and brought to a final volume of 30 mL with RPMI 1640 Thermo Fisher Scientific, Waltham, MA, USA; Cat. No. 11875093). The suspension was centrifuged at 300× *g* for 7 min at RT. To remove red blood cells, the pellet was resuspended in 1 mL of red blood cell lysis buffer and incubated for 1 min with gentle pipetting. The suspension was then diluted to 30 mL with RPMI 1640 and centrifuged under the same conditions to obtain a purified leukocyte pellet. Cells were resuspended in supplemented RPMI [42,43,51], counted using a Neubauer chamber and viability was assessed by Trypan blue exclusion. Splenocytes (2 × 10^5^ cells/well) were cultured and restimulated with BtE, rBlo t 5, rBlo t 21, BTH2, or the mitogen phytohemagglutinin (PHA, Thermo Fisher Scientific, Waltham, MA, USA; Cat. No. 10576-015), as previously described [43,44,51]. Cultures were maintained for either 48 or 72 h, after which supernatants were collected and stored at −20 °C for subsequent cytokine analysis. On the day of supernatant collection, the MTT assay was performed to evaluate the proliferative response of T cells to the treatments and respective stimuli [43,44,51]. The reagent 3-(4,5-dimethylthiazol-2-yl)-2,5-diphenyltetrazolium bromide (MTT, Merck, Rahway, NJ, USA, Cat. No. 475989) was added to each well at a final concentration of 0.6 mg/mL, followed by a 4 h incubation at 37 °C in a humidified 5% CO_2_ atmosphere. After incubation, supernatants were discarded and 150 µL of DMSO (Dinâmica©, São Paulo, SP, Brazil; Cat. No. P1004020000081) was added to dissolve intracellular formazan crystals. Plates were incubated for an additional 15 min, and optical density was measured at 570 nm using a Multiskan GO microplate spectrophotometer (Thermo Fisher Scientific Inc., Waltham, MA, USA). A standard curve generated with purified MTT-formazan (Merck, Rahway, NJ, USA, Cat. No. M2003) was used to calculate the concentration of formazan produced per cell. The stimulation index (SI) was calculated as the ratio of MTT-formazan produced by stimulated cells to that of non-stimulated cells [44,66,67].

### 2.7. Cytokine Quantification in Supernatants

Supernatants from BALF, lung homogenates, and splenocyte cultures were stored at −20 °C until cytokine quantification. The concentrations of IL-4, IL-5, IL-13, IL-10, TGF-β1, TNF, IL-1β, and IFN-γ were measured using commercial ELISA kits, following the manufacturers’ protocols (BD Pharmingen, San Diego, CA, USA; R&D Systems, Minneapolis, MN, USA).

### 2.8. Quantification of IgE, IgG1, IgG2a and IgA Antibodies

Following the administration of anesthetics and before the mice were dead, blood was collected from the axillary vessels via a surgical incision. The abdominal cavity was carefully opened with a C-shaped incision through the skin and abdominal wall, without cutting the peritoneum. The skin was held back with forceps to create a cupped area, and the left vessel were incised with a scalpel to allow blood to pool and be collected. Samples were centrifuged at 13,000× *g* for 10 min at 4 °C to separate the serum, which was aliquoted and stored at −20 °C for subsequent measurement of antigen-specific IgE, IgG1, IgG2a and IgA antibodies, as previously described [42,43,51]. Briefly, indirect ELISA was used to quantify immunoglobulin levels specific to BtE, rBlo t 5, and rBlo t 21. High-binding, half-area 96-well plates (Costar Corning, St. Louis, MO, USA) were coated overnight at 4 °C with 25 µL of BtE (100 µg/mL) or recombinant allergens (5 µg/mL) [42,43,44,51,57]. Plates were then washed at least three times with 1× PBS containing 0.05% Tween-20 (PBS-T) and blocked with PBS-T containing 10% fetal bovine serum (FBS, Bio Nutrientes, Taciba, SP, Brazil; Cat. No. FEO-500) for 1 h at room temperature. After additional washes, serum samples were added to the wells and incubated either for 16 h at 4 °C for the detection of IgE and IgA (diluted 1:10), or for 1 h at 25 °C for the detection of IgG1 and IgG2a (diluted 1:200 and 1:100, respectively, for recombinant allergens; for BtE the dilutions were 1:3200 and 1:250, respectively). Subsequently, HRP-conjugated secondary antibodies anti-mouse IgE (1:500, Abcam, Cambridge, UK, Cat. No. ab99574), anti-mouse IgG1 (1:8000, Abcam, Cambridge, UK, Cat. No. ab97240) and anti-mouse IgG2a (1:1000, Thermo Fisher Scientific, Waltham, MA, USA; Cat. No. M32207) were added and incubated for 1 h at room temperature. For IgA reactivity, we used two steps, incubation for 1h with a biotinylated anti-mouse IgA (1:1000, BD Pharmingen, San Diego, CA, USA; Cat. No. 556978), followed by 30 min incubation with streptavidin-HRP (1:2000, BD Pharmingen, San Diego, CA, USA; Cat. No. 554066). The plates were then washed, and the enzymatic reaction was developed using citrate–phosphate buffer (50 mM, pH 5.0) containing 0.1 mg/mL tetramethylbenzidine (TMB, Amresco, Dallas, TX, USA, Cat. No. 0759-1G) and 2% H_2_O_2_. The reaction was stopped by adding 2 N H_2_SO_4_ (Dinâmica©, São Paulo, SP, Brazil), and absorbance was measured at 450 nm using a Multiskan GO microplate reader (Thermo Fisher Scientific, Waltham, MA, USA). Additionally, the levels of IgA in BALF samples and lung homogenates were assessed using the same indirect ELISA protocol, with the exception that undiluted samples were used during the incubation step.

### 2.9. Evaluation of Blocking Antibodies

An inhibition ELISA was performed to assess whether IgG antibodies from treated mice could block the binding of human IgE to BtE, rBlo t 5, and rBlo t 21 [43]. Plate coating and blocking steps were carried out as previously described. However, the assay involved two sequential incubation steps with serum samples. First, plates were incubated for 2 h at room temperature with individual mouse serum samples from BTH2, Sham, and Control groups. Possible IgE antibodies were inactivated by heat at 56 °C for 1 h [68,69,70]. After washing, a sera pool from allergic human donors [25,43,57] was added and incubated for 16 h at 4 °C. Subsequently, HRP-conjugated anti-human IgE antibodies (Thermo Fisher Scientific, Waltham, MA, USA; Cat. No. A18793) were added and incubated for 1 h at room temperature. Human sera usage was approved by Ethics Commijee of the Maternidade Climatério de Oliveira of Bahia/Federal University of Bahia (MCOB/UFBA), CAAE 25000.013834/2010-96, as part of the project “Risk factors, biomarkers and endophenotypes of severe asthma”.

Detection, reaction termination, and absorbance measurement were conducted as described above. After background subtraction, data were normalized using non-inhibited control wells. The percentage of inhibition was calculated using the formula: 100 − (ISW × 100/UCW), where ISW and UCW represent the absorbance values of inhibited wells (individual mouse sera) and uninhibited control wells, respectively.

### 2.10. Statistical Analysis

Statistical analyses were performed using GraphPad Prism version 8.4.3 (GraphPad Software, San Diego, CA, USA; https://www.graphpad.com/). The Shapiro–Wilk test was used to assess data normality. One-way analysis of variance (ANOVA) followed by Tukey’s post hoc test was used to assess differences between mouse groups when data followed a parametric distribution. For non-parametric datasets, alternative tests recommended by the software were applied. Differences were considered statistically significant when *p* < 0.05.

Ratio analyses were applied in this study to provide a more integrated assessment of immune modulation. A key focus was the antibody ratio, a well-established biomarker for evaluating the efficacy of AIT [71,72]. During AIT, this ratio typically increases, reflecting a long-term shift in the immune response [73,74]. In addition to antibody ratios, leukocyte ratios in BALF were calculated to investigate the potential involvement of lymphocytes and macrophages in the modulation of inflammation. Furthermore, cytokine ratios were analyzed, particularly those comparing Th2-associated cytokines with other cytokines measured in the study. To enable the calculation of ratios involving cytokine concentrations below the detection limit of the assay, values were imputed by assigning the median between zero and the assay’s lower limit of detection, as previously described [43,75]. In some instances, heatmaps were generated to facilitate the visualization of overall data patterns.

Correlation matrix analyses were also performed to identify potential associations between cellular inflammation parameters and cytokine levels (from BALF, lung homogenates, and splenocyte supernatants), as well as between antibody responses and cytokine profiles [74]. Correlation analyses were performed using GraphPad Prism software. For each set of variables, data distribution was first verified with the Shapiro–Wilk test before applying the appropriate correlation test. For example, all total cell counts from the three mouse groups were entered into a column, while the corresponding eosinophil counts were entered into a second column. After performing the normality test, the necessary correlation analysis (Pearson or Spearman) was applied based on the data’s distribution. The resulting correlation coefficients were then organized into a table in Microsoft Excel. This process was repeated for all data pairs to create comprehensive correlation matrices, associating: (i) cell parameters with each other; (ii) antibodies response with each other; (iii) cell parameters with cytokines; and (iv) antibodies response with cytokines. Finally, these correlation coefficients were used to generate colored matrices in R software (version 4.5.1; R Core Team, Vienna, Austria) for statistical computing and effective data visualization.

## 3. Results

### 3.1. Repeated Administration of BTH2 Is Well Tolerated in Mice

As shown in Table 1, serum levels of hepatic enzymes in the BTH2-treated group remained within the reference range for healthy mice, indicating no signs of liver or kidney toxicity. Gamma-glutamyl transferase levels in all groups were below the detection limit and are therefore not shown. Surprisingly, aspartate aminotransferase levels in mice treated with BTH2 were significantly lower than those in the other groups. Furthermore, anatomical analyses revealed no significant alterations in major organs commonly assessed for toxicity, supporting the overall safety of repeated BTH2 administration.

### 3.2. BTH2 Attenuates BtE-Induced Pulmonary Inflammation

**Figure 2:**
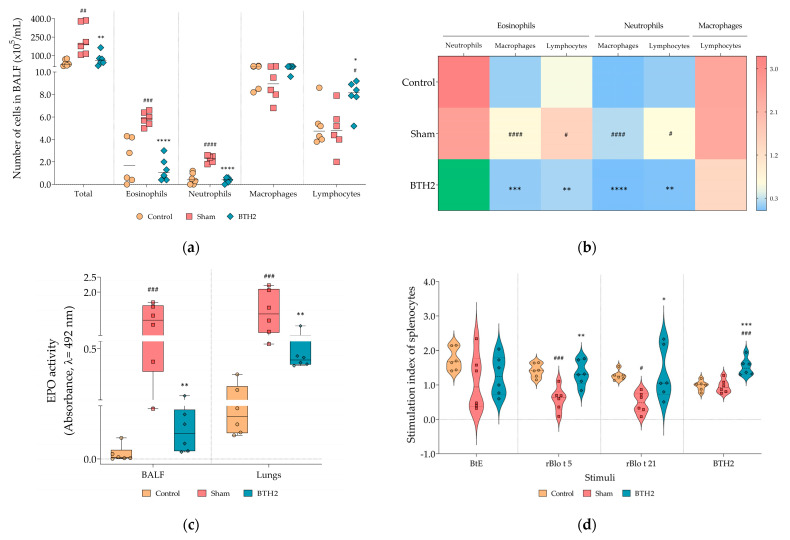
Effect of BTH2 on cellular inflammation and splenocytes proliferation. (**a**) Total and differential cells counting; (**b**) Heatmap analysis using the average values of ratio calculations for the number of specific leucocytes; To enhance color visualization, values exceeding the third quartile of the data distribution were highlighted in green; (**c**) EPO activity in BALF and lungs; (**d**) Stimulation indexes of splenocytes cultured in presence of antigens; BALF, bronchoalveolar lavage fluid; EPO, eosinophil peroxidase activity; BtE, *Blomia tropicalis* extract. One-way ANOVA with post-test of Tukey was used to verify statistical differences between the three mice groups. # Comparison with control. * Comparisons with Sham. *, # *p* ≤ 0.05; **, ## *p* <  0.01; ***, ### *p* <  0.001. ****, #### *p* <  0.0001.

Repeated administration of BTH2 significantly reduced airway inflammation induced by BtE, as evidenced by a marked decrease in total leukocyte counts, eosinophils, and neutrophils in BALF, compared to the Sham group (Figure 2a). Interestingly, BTH2 treatment led to a significant increase in lymphocyte numbers relative to Sham group (Figure 2a). Although macrophage counts in the BTH2 group did not differ significantly from Sham, ratio analyses demonstrated a predominance of lymphocytes and macrophages over eosinophils within the BALF of BTH2-treated mice (Figure 2b), with a stronger statistical significance observed for macrophages. Consistent with the reduction in eosinophilic infiltration, EPO activity was significantly decreased in both lung homogenates and BALF samples from BTH2-treated animals (Figure 2c). The elevated lymphocyte response aligns partially with the splenocyte proliferation data shown in Figure 2d, where restimulation with BTH2 and its parental allergens resulted in a significantly higher SI compared to the Sham group. As observed for BtE (Figure 2d), there were no significant differences among SI of the experimental groups, when splenocytes were stimulated with PHA (Figure S1).

### 3.3. Long-Term Administration of BTH2 Promotes IgG and IgA Production and Suppresses sIgE Responses in Mice

**Figure 3:**
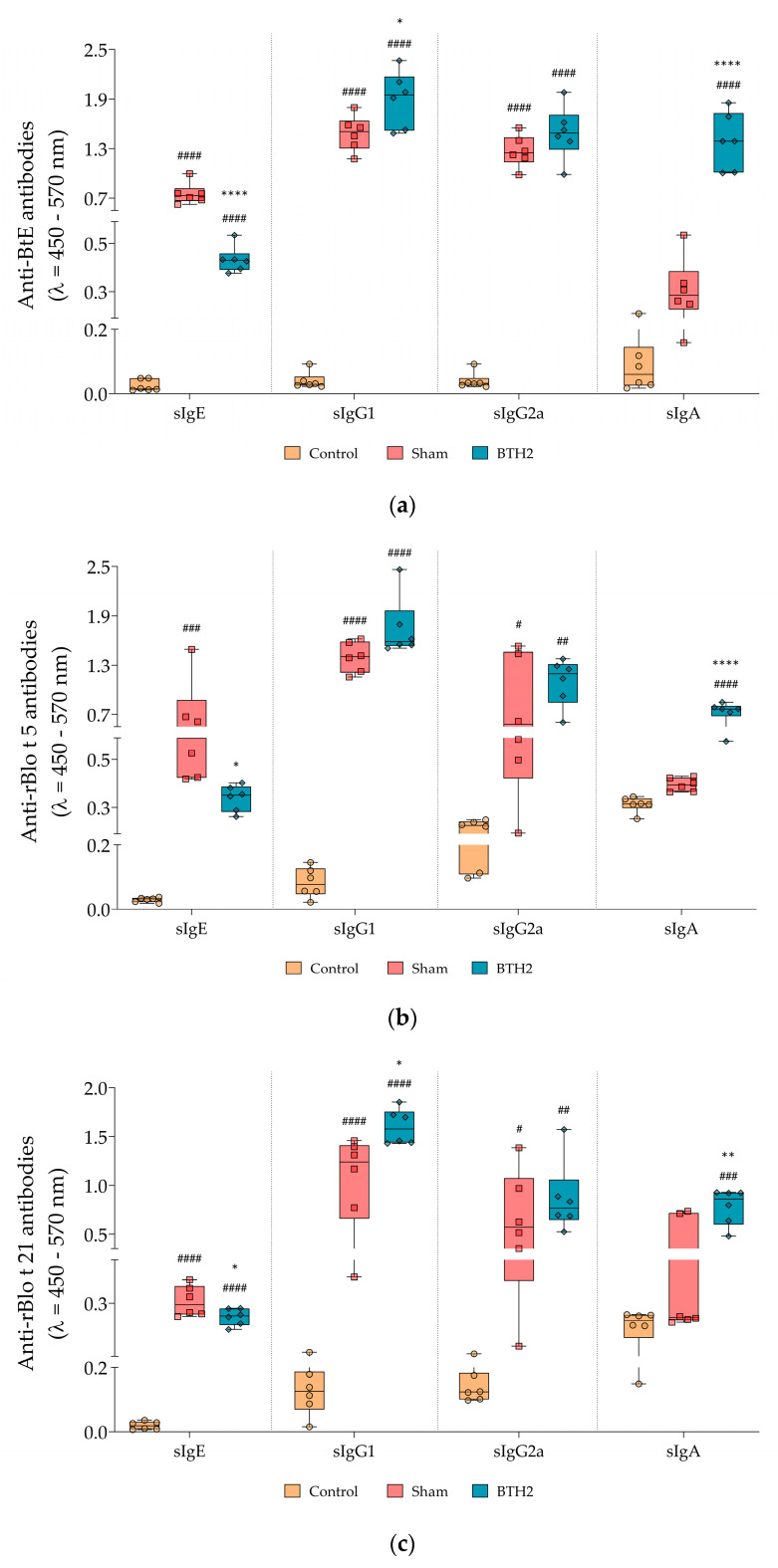
Serological antibody responses induced by long-term BTH2 treatment in a murine model of chronic asthma. Serum levels of specific IgE, IgG1, IgG2a, and IgA against BtE (**a**), rBlo t 5 (**b**), and rBlo t 21 (**c**) were measured by indirect ELISA following repeated BTH2 administration over three months. BtE, *Blomia tropicalis* extract. One-way ANOVA with post-test of Tukey was used to verify statistical differences between the three mice groups. # Comparison with control. * Comparisons with Sham. *, # *p* ≤ 0.05; **, ## *p* <  0.01; ### *p* < 0.001. ****, #### *p* <  0.0001.

Mice treated with BTH2 displayed a significant reduction in specific IgE (sIgE) against BtE and the major allergens rBlo t 5 and rBlo t 21, compared with the Sham group (Figure 3a–c). The specific IgG1 (sIgG1) against all antigens increased following treatment with the hypoallergen; however, this increase did not reach statistical significance for rBlo t 5 (Figure 3a–c), when compared to Sham group. A non-significant trend toward increased sIgG2a levels was observed for all antigens (Figure 3a–c). In contrast, serum IgA levels against both the parental allergens and BtE were significantly elevated in BTH2-treated mice in comparison with mice from Sham group (Figure 3a–c).

### 3.4. Prolonged Subcutaneous Administration of BTH2 Induces Mucosal IgA and Elicits Systemic IgG-Dominated Responses, Which Blocks IgE–Allergen Interactions

**Figure 4:**
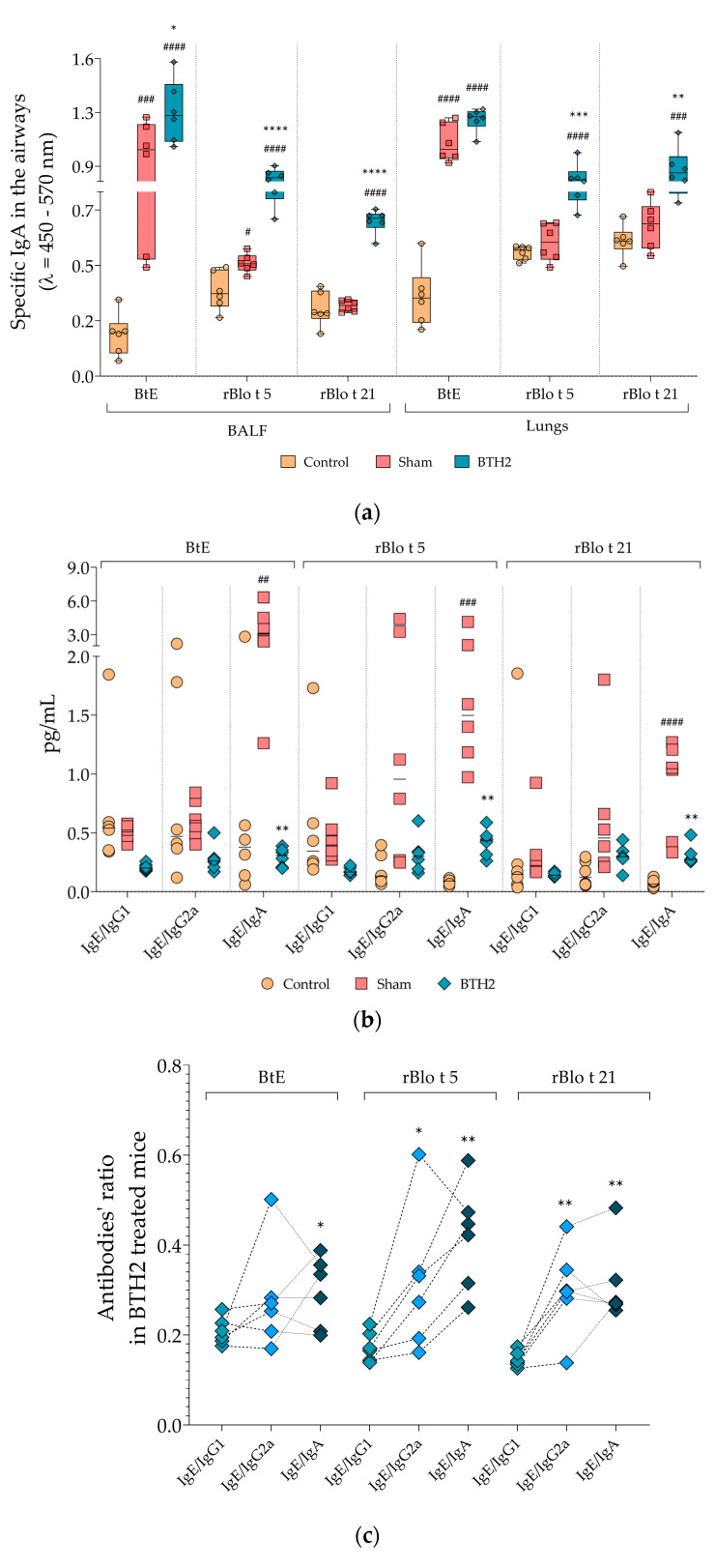
Mucosal and systemic antibody responses and ratio analyses following long-term BTH2 treatment in a murine model of chronic asthma. (**a**) Serum levels of specific IgA against BtE, rBlo t 5, and rBlo t 21 were measured by indirect ELISA after repeated subcutaneous administration of BTH2 over a three-month period. (**b**) Ratio analyses for the measured antibodies. Statistical differences between the three experimental groups were analyzed using one-way ANOVA followed by Tukey’s post hoc test. # Comparison with control. * Comparisons with Sham. *, # *p* ≤ 0.05; **, ## *p* <  0.01; ***, ### *p* <  0.001. ****, #### *p* < 0.0001. (**c**) Tukey’s multiple comparisons test was also used to assess differences in serological antibody ratios of BTH2-treated mice. * Ratio comparisons with IgE/IgG1. * *p* ≤ 0.05; ** *p* <  0.01. BALF, bronchoalveolar lavage fluid; BtE, *Blomia tropicalis* extract.

As displayed in Figure 4a, prolonged subcutaneous administration of BTH2 led to a significant increase in antigen-specific IgA levels in the airways. All antigens tested exhibited elevated IgA levels in BALF and lung homogenate samples, apart from BtE in lung homogenates, which did not reach statistical significance. Figure 4b illustrates the ratios of serological antibodies. While the IgE/IgG1 and IgE/IgG2a ratios in the BTH2 group did not reach statistical significance compared with the Sham group—likely due to high variability in the Control and Sham data—the IgE/IgA ratio showed a significant reduction in BTH2-treated mice for all antigens. Although this represents a surprising and intriguing finding, further subclass ratio analyses confirmed that the systemic humoral response remained predominantly IgG-mediated, particularly through IgG1, as evidenced by the significantly lower IgE/IgG1 ratio compared with other antibody subclass ratios in BTH2-treated animals (Figure 4c).

### 3.5. Repeated Subcutaneous Administration of BTH2 Induces Antibodies That Blocks IgE–Allergen Interactions

**Figure 5:**
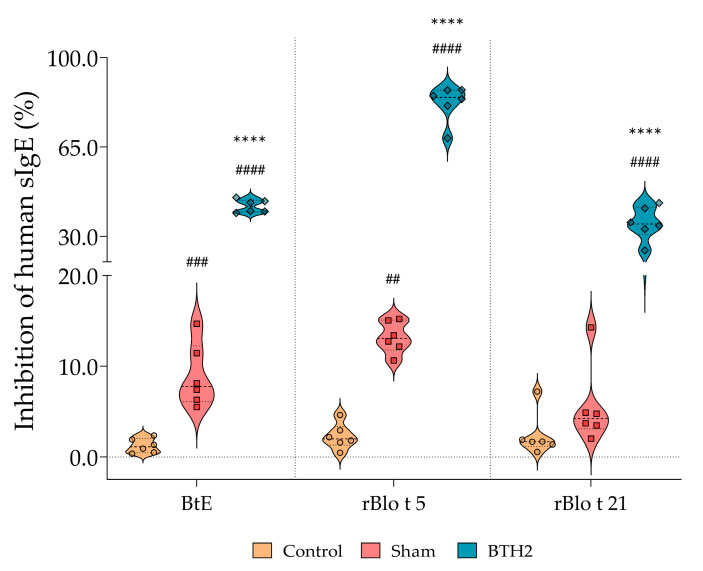
Inhibition of human IgE binding to BtE and parental allergens by sera derived from treated and non-treated mice. The percentage of inhibition was used to assess the blocking capacity of non-IgE serum antibodies. Statistical differences between the three experimental groups were analyzed using one-way ANOVA followed by Tukey’s post hoc test. # Comparison with control. * Comparisons with Sham. ## *p* <  0.01; ### *p* <  0.001; ****, #### *p* <  0.0001. All mouse sera were heat-inactivated prior to testing in order to eliminate residual IgE activity.

The immune response induced by repeated dosage of BTH2 was sufficient to inhibit IgE–allergen interactions. Importantly, mouse sera used in these inhibition assays were heat-inactivated prior to testing, thereby excluding any potential contribution of murine IgE and confirming that the blocking effect derives from heat-resistant antibodies (mainly IgG and IgA). In fact, when BtE, rBlo t 5 and Blo t 21 were immobilized on ELISA plates, pre-incubation with sera from BTH2-treated mice significantly reduced the IgE-binding capacity of human allergic sera (Figure 5).

### 3.6. Treatment with Repeated Doses of BTH2 Downmodulates Th2 Cytokines and May Promote Th1-Biased and/or Regulatory Immune Responses

**Figure 6:**
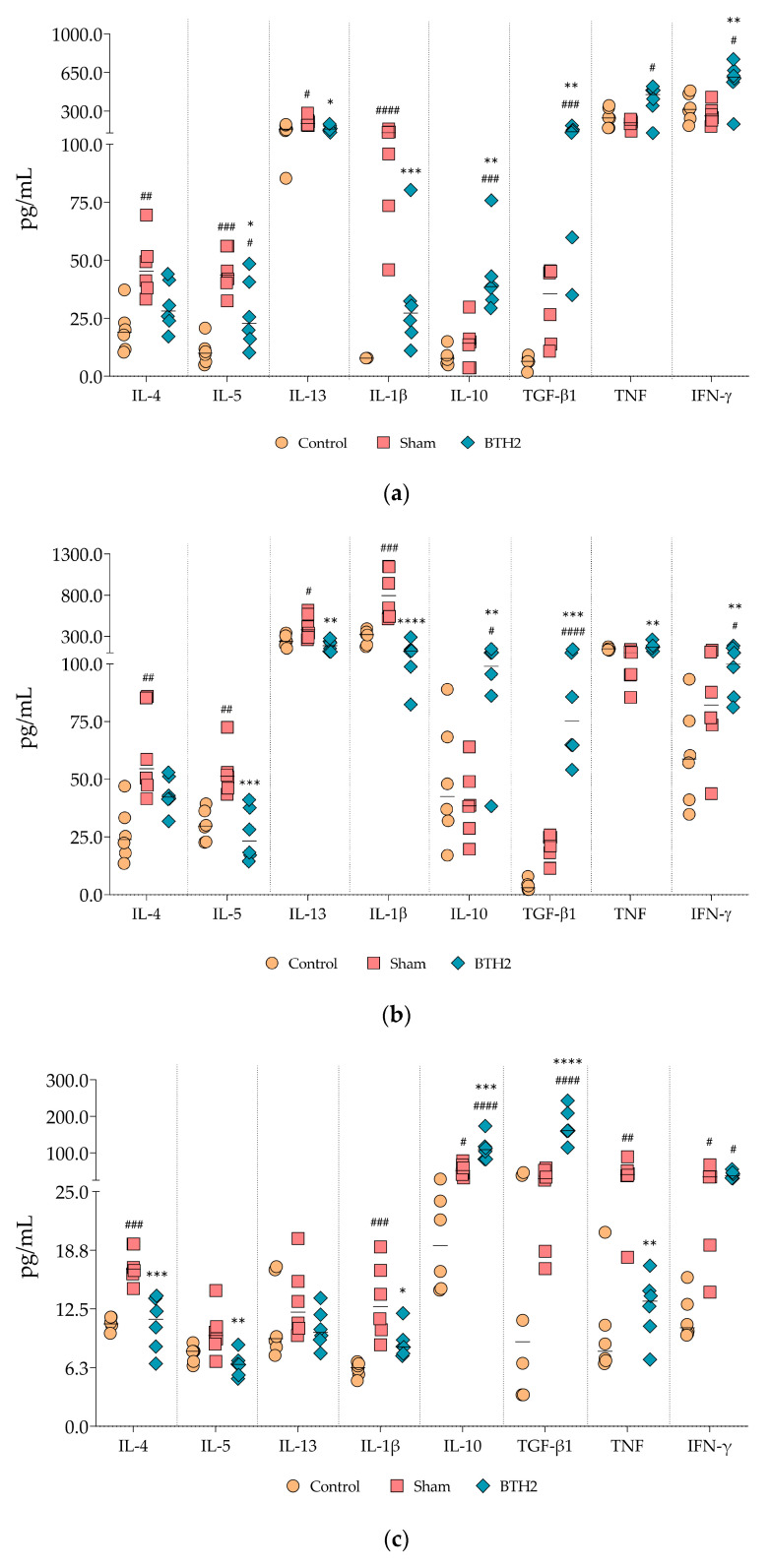
Effect of long-term BTH2 treatment in cytokines levels in the airway and spleen of mice. Cytokines were measured in BALF (**a**), lung homogenates (**b**), and unstimulated splenocytes (**c**) were measured by indirect ELISA following repeated BTH2 administration over three months. BALF, bronchoalveolar lavage fluid. One-way ANOVA with post-test of Tukey was used to verify statistical differences between the three mice groups. # Comparison with control. * Comparisons with Sham. *, # *p* ≤ 0.05; **, ## *p* <  0.01; ***, ### *p* <  0.001. ****, #### *p* <  0.0001.

Cytokine quantifications were performed using samples collected at the time of euthanasia, including BALF, lung homogenates, and spleens. For the latter supernatants from unstimulated and antigen-stimulated splenocyte cultures were analyzed by ELISA. All three types of samples are shown in Figure 6 and Figures S2–S6.

A marked reduction in Th2-associated cytokines (IL-4, IL-5 and IL-13) was observed in the airways (Figure 6a,b) and splenocyte supernatants (Figure 6c) of allergic and BTH2-treated mice compared to the allergic and Sham-treated group. Notably, although a trend toward reduced IL-4 presence was detected in BALF and lung homogenate tissue, this reduction did not reach statistical significance (Figure 6a,b). In contrast, different from what occurred for IL-5 and IL-13, the suppression of IL-4 secretion by splenocytes was statistically significant (Figure 6c).

IL-1β levels exhibited a consistent and significant decrease across all sample types (Figure 6a–c), indicating a broader anti-inflammatory effect of BTH2 treatment. Additionally, the usually anti-inflammatory cytokines IL-10 and TGF-β were significantly upregulated in all samples analyzed (Figure 6).

Regarding Th1-associated cytokines, both TNF and IFN-γ levels were elevated in airway samples (Figure 6a,b), although only IFN-γ reached statistical significance. Interestingly, TNF secretion by splenocytes was significantly reduced in BTH2-treated mice compared to Sham, suggesting a tissue-specific modulation of this cytokine. IFN-γ secretion by splenocytes, while increased, did not reach statistical significance.

Stimulation of splenocytes with BTH2 did not substantially alter the overall cytokine profile compared to unstimulated cells but statistically decreased IL-13 and increased IFN-γ levels compared to the Sham group (Figure S2). Stimulation with BtE, the parental allergens of BTH2, and PHA yielded cytokine secretion profiles comparable to those observed with BTH2 stimulation (Figures S3–S6). IL-1β and TGF-β were the two cytokines that behave consistently throughout all stimuli, with down- and up-regulation, respectively (Figure 6c and Figures S2–S6).

### 3.7. Correlation Matrix Analyses Support the Involvement of Cytokines in Key AIT Markers

To obtain an integrated view of the relationships between cellular, antibody, and cytokine parameters across all three sample types (BALF, lung homogenate, and splenocyte supernatants), we conducted correlation analyses and visualized the results using correlation matrices. It is important to point out that correlation analyses should be interpreted as indicators of immunological associations rather than absolute antibody predominance.

**Figure 7:**
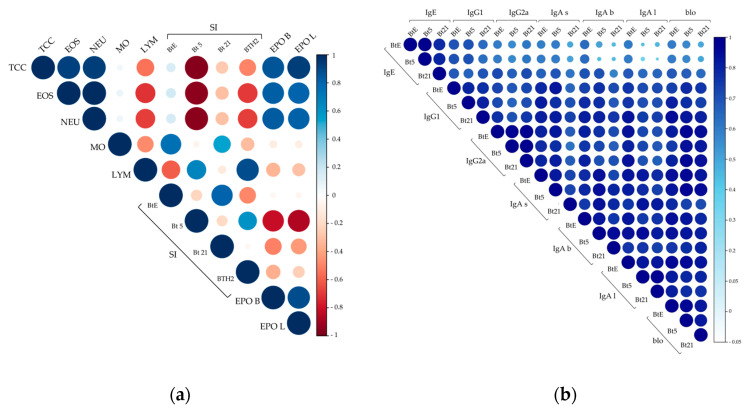
Primary correlation matrix analyses. (**a**) Correlation matrix of effector cell-related parameters; (**b**) Correlation matrix of antibody responses to *Blomia tropicalis* extract (BtE), rBlo t 5 (Bt5), and rBlo t 21 (Bt21). Pearson or Spearman correlation coefficients were calculated based on data distribution. Color intensity and circle size represent at the same time the strength and direction of correlations. For example, dark blue and larger circles indicate a positive and strong correlation close to 1, while intense red color and larger circles indicate a strong negative correlation (see the scale on the right). TCC, total cells count; EOS, eosinophils; NEU, neutrophils; MO, macrophages; LYM, lymphocytes; SI, stimulation index; EPO, eosinophil peroxidase, with letters “b” and “l” for BALF and lungs samples, respectively; for IgA antibodies, letters “s”, “b” and “l” mean IgA antibodies measured in sera, BALF and lungs, respectively; blo, blocking antibodies.

As shown in Figure 7a, cellular parameters exhibited strong positive correlations among eosinophils, neutrophils, total cell counts, and EPO levels in both BALF and lung tissue. These correlations reflected the consistent nature of the allergic response within each experimental group—characterized by either uniformly low or high levels of inflammatory cells and EPO. Interestingly, lymphocyte counts displayed moderate to strong negative correlations with eosinophils, neutrophils, and total cell counts, suggesting that elevated lymphocyte presence may be associated with a reduced influx of pro-inflammatory granulocytes, potentially indicating an immunomodulatory role. Surprisingly, SI values for BTH2—and especially for rBlo t 5—were from moderate to strong negative correlations with eosinophils, neutrophils, and total cell counts. These antigens’ proliferative response may inversely correlate with airway inflammation intensity. Figure 7a also shows a strong positive correlation between lymphocyte numbers and SI for BTH2. Notably, the SI for rBlo t 5 also exhibited a strong negative correlation with EPO levels, suggesting splenic cells may modulate eosinophil-driven inflammation.

Figure 7b represents the matrix for antibody measurements with a consistent positive correlation throughout the data. When assessing correlations among sIgG subclasses, moderate to strong positive correlations were observed. Notably, sIgG2a showed stronger positive correlations with IgA antibodies than sIgG1 did. While correlations between sIgE and both sIgG2a and sIgA were generally weak, sIgG1 displayed moderate to strong positive correlations with sIgE, suggesting that a portion of the sIgG1 response may remain Th2-polarized.

When focusing on blocking antibodies, the strongest positive correlations were observed between sIgG2a and sIgA, rather than with sIgG1 (Figure 7b). This finding contrasts with the results displayed in Figure 4b, which indicated IgG1 as the primary blocking antibody.

**Figure 8:**
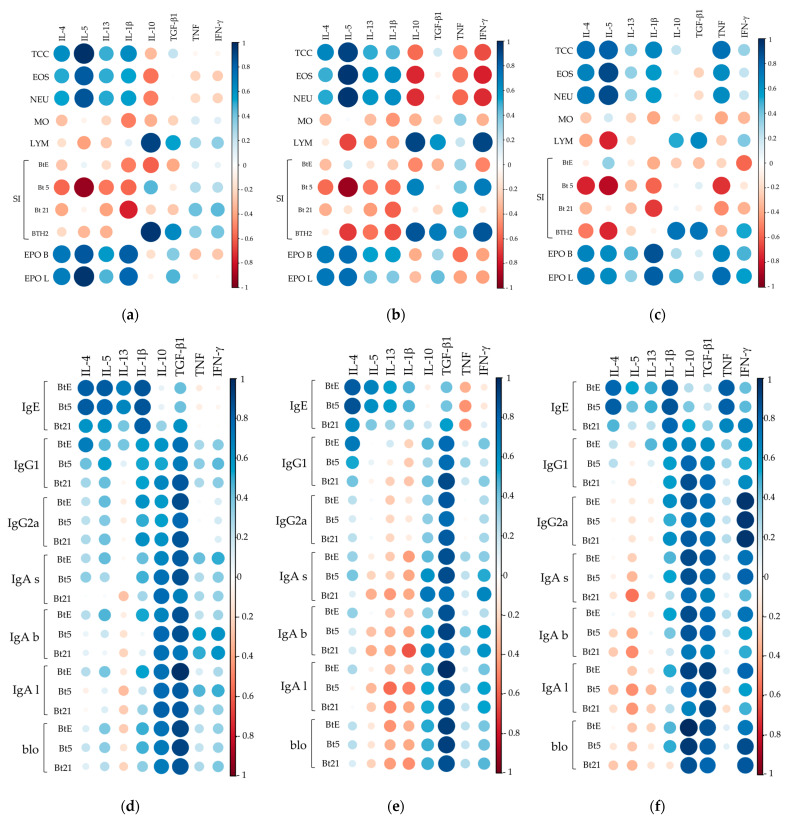
Correlation matrices between cytokine levels and cellular or humoral immune parameters. Correlation matrices between effector cell-related parameters and cytokine levels in BALF (**a**), lung homogenates (**b**), and splenocyte supernatants (**c**). Correlation matrices between cytokine levels and antibody responses to *Blomia tropicalis* extract (BtE), rBlo t 5 (Bt5), and rBlo t 21 (Bt21) in BALF (**d**), lung homogenates (**e**), and splenocyte supernatants (**f**). Pearson or Spearman correlation coefficients were applied depending on data distribution. Color intensity and circle size represent the strength and direction of correlations. TCC, total cells count; EOS, eosinophils; NEU, neutrophils; MO, macrophages; LYM, lymphocytes; SI, stimulation index; EPO, eosinophil peroxidase, with letters “b” and “l” for BALF and lungs samples, respectively; for IgA antibodies, letters “s”, “b” and “l” mean IgA antibodies measured in sera, BALF and lungs, respectively; blo, blocking antibodies.

As shown in Figure 8, several consistent correlations were observed between Th2 cytokine levels in BALF, lung homogenates, and splenocyte supernatants and various cellular parameters. IL-5 showed the strongest positive correlations with total cell counts, eosinophils, and neutrophils in all samples (Figure 8a–c), along with moderate-to-strong correlations with EPO activity. Conversely, SI for rBlo t 5 and BTH2 exhibited strong negative correlations with IL-5, particularly in lung and splenocyte samples (Figure 8a,c). IL-4 and IL-1β levels were strongly positively correlated with EPO activity, especially in BALF, with IL-1β showing even stronger associations in BALF and splenocytes (Figure 8a,c).

Regarding IL-10, it showed significant negative correlations with eosinophil and neutrophil counts only in lung samples (Figure 8b), while BALF and lung IL-10 levels displayed strong positive correlations with lymphocyte counts and BTH2 SI (Figure 8a,b). Although IFN-γ correlations in BALF and splenocyte supernatants were mostly weak, lung values resembled IL-10’s, with slightly stronger positive/negative trends (Figure 8b). TGF-β exhibited weak-to-moderate correlations, including moderate links to lymphocyte counts and BTH2 SI across samples. Splenocyte TNF levels strongly correlated with EPO activity as well as with total, eosinophil, and neutrophil counts (Figure 8c).

In the context of antibody responses, IL-4 levels in airways and splenocyte supernatants strongly correlated with IgE against BtE and rBlo t 5 but only weakly to moderately with rBlo t 21 (Figure 8d–f). In contrast, IL-1β in BALF and splenocyte supernatants strongly and positively correlated with sIgE. TNF showed weak-to-moderate sIgE correlations in airways (Figure 8d,e) but a strong positive correlation in splenocytes (Figure 8f), suggesting tissue-specific immune responses. Splenocyte IFN-γ levels were from moderate to strong correlated with antibody levels, especially sIgG2a, and with blocking antibodies against BTH2-parental allergens (Figure 8f).

Unexpectedly, no consistent negative correlations were observed between IgG1, IgG2a, or IgA and Th2 cytokines (Figure 8d–f). The pattern was as follows: (i) in BALF samples, weak positive correlations (IL-4, IL-5, and IL-1β) or weak negative correlations (IL-13) were seen with IgG1, IgG2a, IgA, and blocking antibody measurements (Figure 8d); (ii) in lung homogenates, correlations shifted to mostly negative for IL-5, IL-13, and IL-1β, while some positive correlations with IL-4 remained (Figure 8e); and (iii) in splenocyte cultures, correlations between cytokines and non-IgE antibodies or blocking antibodies were generally weak, either positive (IL-1β) or negative (Th2 cytokines) (Figure 8f).

The typically anti-inflammatory cytokine IL-10 showed weak-to-moderate correlations with non-IgE antibodies in airways (Figure 8d,e), except for strong positive correlations with BALF IgA in BALF and lung homogenates (Figure 8e). In contrast, splenic IL-10 strongly correlated with a few antibodies, particularly blocking antibodies (Figure 8f). Similarly, splenocyte-derived IL-10 exhibited strong correlations with select antibody responses, especially blocking antibodies (Figure 8f). TGF-β levels consistently demonstrated moderate-to-strong positive correlations with IgG1, IgG2a, IgA, and blocking antibodies across all sample types (Figure 8d–f).

The ratio analysis, combined with correlations involving cellular parameters and specific antibody measurements, allows us to infer potential influences of certain cytokines on the modulation of others. For example, in Figures S7–S9, when examining the IL-5/IL-10 ratio pattern in BALF and lung samples and its correlation with lymphocyte counts, a strong negative correlation was observed. This suggests that IL-10—likely produced by these lymphocytes—is present in much higher amounts than IL-5, indicating that the observed modulatory pattern is probably driven by IL-10. Another notable finding from the cytokine ratio correlation analysis is the nearly uniform pattern of negative correlations, mostly moderate to strong, between the cytokine ratios and the SI for BTH2 (Figures S7–S9).

In the correlations between cytokine ratios and antibody responses, a clear potential role for TGF-β was observed in the modulation of IL-4 and IL-13. Almost all measured antibodies, especially non-IgE isotypes, showed moderate to strong positive correlations with cytokine ratios in BALF and lung samples (Figures S10 and S11). Surprisingly, the splenocyte cytokine profile revealed moderate to strong correlations for the ratio of Th2 cytokines and IL-10, TGF-β, and also IFN-γ (Figure S12), suggesting that a mixed immune profile may be associated with the improvement of the asthmatic condition and the induction of positive markers for AIT. Interestingly, the strong positive correlations between measurements of IgE antibodies and TNF were strengthened by ratio analysis, since in splenic samples strong negative correlation between the ratio Th2 cytokines/TNF were observed, indicating that TNF was much more secreted by splenocytes (Figure S12) and maybe related to allergic inflammation, although the presence of this cytokine in the airways was negatively correlated to leucocytes counts.

## 4. Discussion

Mimicking asthma by using chronic exposure to an allergen source is pivotal to better evaluating hypoallergenic derivatives for AIT. The present study employed a clinically relevant chronic asthma model induced by *Blomia tropicalis*, a major source of indoor allergens in tropical regions and a significant contributor to allergic asthma in affected populations [26,43]. This approach provides greater translational relevance compared to traditional ovalbumin-based models [78,79], which, despite being widely used and scientifically useful to a degree, lack clinical correlation to human allergic asthma [80,81,82]. Our group, with the intend of better mimic this illness induced by an airborne allergen source and verify the possibility of AIT for asthmatic patients, has developed a model using the mite *Dermatophagoides pteronyssinus* [51]. In this study, this murine model was adapted to a chronic exposure to *Blomia tropicalis* soluble allergens. This approach allows for the induction of sustained airway inflammation and remodeling, better reflecting the pathophysiological features of human disease, as we previously demonstrated [51]. Importantly, our study also evaluates the repeated subcutaneous administration of BTH2—a hypoallergenic derivative of major *Blomia tropicalis* allergens—not only as a therapeutic intervention but also as a preclinical model to assess the safety of long-term AIT.

Regarding safety, repeated BTH2 administration in the chronic asthma model did not induce toxicity. Although aspartate aminotransferase levels were significantly lower in treated mice, a definitive protective hepatic effect cannot be confirmed, as alanine aminotransferase levels remained unchanged. Notably, since macro-aspartate aminotransferase complexes (the enzyme bound to immunoglobulins) have been reported in patients undergoing AIT [83]. However, the consistently low AST levels observed in BTH2-treated mice may indicate the absence of such complexes. This suggests that future BTH2-based AIT in humans is unlikely to trigger this secondary effect, potentially resulting in a cleaner liver enzyme profile, especially when combined with the reduced systemic inflammation demonstrated in this study.

Prolonged treatment of asthmatic mice indeed reduced several allergenic markers in the present study. The reduced number of total leucocytes and especially neutrophils and eosinophils in the mice’s airway was, in fact, an important finding. Severe asthma can be often associated with both eosinophilic and/or neutrophilic infiltration in the lungs [84,85,86,87,88]. The observed reduction in these cell types in our model may be linked to the increased presence of lymphocytes and macrophages, which are known to produce cytokines such as IL-10, TGF-β and IFN-γ that may modulate the recruitment of leucocytes [34,40,47,89,90,91,92]. These findings are an improvement for BTH2 candidate, especially when we compared the data from our findings for the acute murine model, which showed only a possible role of macrophages in the downregulation of inflammatory response [43]. Additionally, several analyses in the current chronic study support the influence of regulatory cytokines in modulating inflammation. Indeed, previous studies have shown that immunoregulatory effects may be mediated, in part, by IL-10-producing antigen-presenting cells, including macrophages and dendritic cells [89,90,91]. Additionally, our correlation analyses reinforce the potential role of increased airway lymphocytes in regulating allergic inflammation. Notably, prior clinical studies demonstrated that IL-10-producing T regulatory (Treg) cells persist at high frequencies for up to three years during house dust mite AIT [93,94]—a finding consistent with the strong correlation observed between lymphocyte numbers and IL-10 levels in the airways of BTH2-treated mice.

Another important indicator of reduced airway inflammation in asthmatic mice was the decreased EPO activity, which was consistent with the lower eosinophil counts and the observed reduction in IL-5 levels in the airways of treated mice. IL-5 is essential to the pathogenesis of allergic disease, especially acting on the differentiation, recruitment, survival, and degranulation of eosinophils [95]. This cytokine downregulation in our model was an important finding, especially when a clinical trial with a hypoallergenic derivative has shown how a subcutaneous AIT schedule was dependent on a decrease in IL-5-secreting T cells [47,48]. Moreover, the importance of EPO for the pathogenesis and progression of allergic asthma is well established [96,97,98]. The enzyme is one of the products of eosinophil degranulation, producing oxidative species that cause tissue damage and contribute to bronchial remodeling [96,97]. Furthermore, previous studies revealed how EPO contributes to the induction of asthmatic profile in mice, through protein carbamylation [97]. We also found the decrease in this triad in acute experimental models from our group [42,43,64], as well as in our previous chronic asthma model using another hypoallergen for treatment of mice [51]. However, we should reinforce that our study was limited concerning a functional respiratory assessment, which could be better evaluated by plethysmography experiments.

To support the role of T cell priming in IL-5 downregulation and asthma symptom improvement, the SI for rBlo t 5 and BTH2 negatively correlated with IL-5, eosinophil counts, and EPO activity. rBlo t 5 SI showed strong negative correlations with this triad, suggesting reduced pathogenic responses upon re-exposure with a major *B. tropicalis* allergen. Although weaker, BTH2 SI also negatively correlated with these markers, demonstrating preserved immunogenicity with reduced allergenicity—a key AIT goal. An effective hybrid AIT vaccine should enhance or maintain T cell proliferation similar to its parental allergens [31,32,47,48,49]. While our SI data seem to indicate the induction of this hall mark by BTH2, we should acknowledge another limitation of our study: the lack of flow cytometry analyses to further characterize the T cell and macrophage subsets induced by repeated BTH2 administration. 

The antibody response following AIT is a critical indicator of treatment efficacy. One of the main objectives is the induction of blocking antibodies—particularly IgG and IgA –alongside the gradual reduction in IgE production [33,36,37,38,39]. Fulfilling this aim, repeated exposure to BTH2 in asthmatic mice led to a significant reduction in IgE levels, likely associated with decreased IL-4 and IL-1β expression. Concurrently, elevated IFN-γ levels were observed, aligning partially with an increase in IgG2a production—an antibody typically associated with Th1 or regulatory immune responses. Such results were not observed in previous acute models used by our research group [42,43], highlighting the relevance of this chronic exposure approach [51]. Interestingly, IgG1 levels remained high, a finding consistent with other murine studies involving hypoallergens as therapeutics or immunizers [41,42,43,44,68,99]. This robust IgG response, especially the generation of potential blocking antibodies capable of binding to parental allergens and preventing their interaction with effector cells, represented a favorable outcome [31,32,33,34]. This immunoglobulin profile supports the notion that BTH2 not only reduces allergenicity but also enhances humoral blocking responses—key attributes of an effective AIT formulation. Furthermore, while we acknowledge that a simultaneous competitive ELISA (co-incubating murine sera and human IgE) would provide an even more stringent demonstration of blocking activity, this could not be performed because no serum samples remain available, as all aliquots had already been processed and consumed for multiple antibody and inhibition assays. We recognize this as a limitation and will prioritize such competitive assays in future studies.

Unexpected, the robust IgA response observed in this study—especially considering the subcutaneous route used—was not unwelcome. Antigen-specific IgA antibodies were consistently detected in both the airways and serum of BTH2-treated mice, and their levels showed strong correlations with TGF-β concentrations. While previous studies have emphasized the sublingual route as the most effective for inducing mucosal IgA during AIT [71,100,101], accumulating evidence indicates that subcutaneous immunotherapy can also promote IgA production [93,102,103]. Notably, a clinical study has shown that IgA antibodies elicited by AIT using the subcutaneous route were correlated with TGF-β levels detected in nasal secretions [102]—a finding that closely parallels our results. Despite involved in dual roles in allergy [104,105], TGF-β can induce the generation of regulatory T cells and directly participates in the suppression of effector T cell functions [102,104,106]. In addition, the isoform measured in the present study displayed a protective role against airway hyperresponsiveness and asthma exacerbations [107,108]. In fact, significantly higher levels of exhaled TGF-β1 have been observed in children with controlled asthma compared to those experiencing exacerbations [107]. Furthermore, a subset of Treg expressing TGF-β has been shown to contribute to the re-establishment of peripheral tolerance to allergens during AIT [106].

Cytokine data of the present article reinforce some findings. IL-4, reduced after repeated exposure of BTH2, is central to sensitization mechanisms, promoting Th2 differentiation and class switching to IgE in B cells [109]. It also contributes to the activation of eosinophils and mast cells, key players in allergic inflammation [110,111]. IL-13 plays a dual role—participating in IgE production but, more importantly, mediating mucus hypersecretion during the effector phase of allergic inflammation [109,110,111,112]. Recent studies have linked IL-13 to chronic allergic diseases, underscoring its role in localized inflammation [113,114]. Unfortunately, another limitation of our study is the absence of histopathological lung analyses, which will be essential in future investigations to determine whether the observed IL-13 reduction is linked to decreased mucus production, as seen in our previous murine models [42,43,51], and also to assess whether tissue remodeling was also prevented, as demonstrated in our earlier chronic AIT model [51]. IL-1β, which can be secreted by B cells, supports their activation and differentiation into plasma cells, enhancing IgE production [115]. Its levels in the present study corroborates previous findings, since there was a strong correlation with IgE in all types of samples in the present study. Emerging evidence suggests that IL-1β may serve as a serum biomarker for identifying severe and persistent cases of allergic rhinitis and asthma [116,117] and contributes to allergic inflammation by promoting neutrophil recruitment, airway hyperresponsiveness, and mucus secretion [118,119].

TNF measurements in this study revealed paradoxical behavior. In splenocyte supernatants, reduced levels were positively correlated with IgE antibodies, whereas in the airways, increased levels were negatively correlated with allergenic markers. TNF plays a known role in allergic sensitization and is typically elevated during late-phase allergic reactions, particularly by promoting neutrophil recruitment [109]. Interestingly, some studies have proposed a pleiotropic role for TNF, including a potential immunoregulatory function [120], by activating regulatory T cells, tolerogenic dendritic cells, and myeloid-derived suppressor cells [120,121]. However, further studies assessing TNFR2 expression will be necessary to determine whether TNF production by other cells contributes to enhanced Treg differentiation and function, as previously reported [122].

The partial IFN-γ–mediated suppression observed in our study aligns with its known roles in allergy, including promotion of Th1 polarization, reduced allergen presentation, inhibition of Th2 cytokine secretion, eosinophil migration and survival, and blockade of IgE class switching [123,124]. Previous studies with hypoallergens have found a switch in the allergenic response due to the Th1 polarization and IFN-γ production [42,125]. Nevertheless, a recent clinical trial with a recombinant hypoallergen detailed how this induction of Th1 response in AIT is not always ideal, since this profile failed to produce IgG4 blocking antibodies functions [126].

Our results, on the other hand, indicate a mixed immune profile, highlighting predominance of a regulatory one, with high levels of TGF-β and IL-10. The latter can be considered an anti-inflammatory cytokine [127] and its increase in AIT is considered a parameter of formulation efficacy, as IL-10 can promote class switching from IgE to IgG4 in humans, which is an important marker of tolerogenesis and acts as a blocking antibody of IgE-allergens interactions [48,49,128,129].

## 5. Conclusions

Considering all data together, repeated subcutaneous administration of the hypoallergenic candidate BTH2 in a murine model of asthma induced by *Blomia tropicalis* resulted in a broad immunomodulatory profile consistent with effective AIT, along with a favorable safety profile. The treatment significantly attenuated key features of allergic airway inflammation while promoting hallmark indicators of successful AIT—most notably, the induction of mixed immunoregulatory and Th1-associated cytokines such as IL-10, IFN-γ, and TGF-β. These cytokines showed correlations with protective antibody responses, including IgA, IgG2a, and blocking antibodies. Correlation matrix analyses further suggest that the antibody profile induced may reflect a higher influence of a regulatory immune mechanisms rather than a classical Th1 response, but both seemed to participate in the final response deviated away from a Th2-dominated allergic phenotype. Therefore, BTH2 remains a promising hypoallergenic derivative that warrants further investigation in clinical settings, including potential trials involving asthmatic patients.

## Figures and Tables

**Table 1:** Serum hepatic and renal enzyme levels and organ weights in mice following repeated administration of BTH2.

Parameter	Mean ± SD ^1^	*p*-Values
Alanine aminotransferase (U/L) ^2^		
Control	63.05 ± 0.34	--
Sham	60.41 ± 9.77	0.9498 ^#^.
BTH2	60.23 ± 11.15	0.9429 ^#^; 0.9997 *.
Aspartate aminotransferase (U/L) ^3^		
Control	148.21 ± 1.72	--
Sham	149.54 ± 4.29	0.9089 ^#^.
BTH2	96.05 ± 3.13	0.0011 ^#^; 0.0010 *.
Uric acid (mg/dL) ^4^		
Control	4.26 ± 0.60	--
Sham	5.14 ± 1.17	0.5405 ^#^.
BTH2	3.20 ± 0.29	0.4433 ^#^; 0.1575 *.
Liver weight (mg)		
Control	994.72 ± 135.67	--
Sham	1035.12 ± 229.73	0.9071 ^#^.
BTH2	1157.92 ± 101.96	0.2362 ^#^; 0.4265 *.
Spleen weight (mg)		
Control	108.33 ± 33.35	--
Sham	126.78 ± 59.82	0.7326 ^#^.
BTH2	85.83 ± 23.49	0.6290 ^#^; 0.2405 *.
Kidneys weight (mg)		
Control	338.03 ± 21.83	--
Sham	293.83 ± 29.33	0.0757 ^#^.
BTH2	322.85 ± 42.15	0.6995 ^#^; 0.2929 *.
Lungs weight (mg)		
Control	53.50 ± 22.83	--
Sham	76.50 ± 44.40	0.4704 ^#^.
BTH2	62.33 ± 28.39	0.8901 ^#^; 0.7441 *.

^1^ standard deviation; normal range were based in the previous literature and similar methods of measurements; ^2^ 26.30–80.00; ^3^ 41.80–137.30; ^4^ 2.52–6.72 [76,77]; *n* = 6, for each mice group; one-way ANOVA with post-test of Tukey was used to verify statistical differences between the three mice groups. # *p*-value compared to Control; * *p*-value compared to Sham. Differences were considered statistically significant when *p* < 0.05 and were highlighted in bold.

## Data Availability

The data presented in this study are available on request from the corresponding authors.

## Supplementary Materials

The following supporting information can be downloaded at: https://www.mdpi.com/article/10.3390/biomedicines13112657/s1. Figure S1: Stimulation indexes of splenocytes cultured in presence of phytohemagglutinin. Figure S2: Effect of long-term BTH2 treatment in cytokines levels of *B. tropicalis* extract-stimulated splenocytes. Figure S3: Effect of long-term BTH2 treatment on cytokines levels of BTH2-stimulated splenocytes. Figure S4: Effect of long-term BTH2 treatment in cytokines levels of rBlo t 5-stimulated splenocytes. Figure S5: Effect of long-term BTH2 treatment on cytokines levels of rBlo t 21-stimulated splenocytes. Figure S6: Effect of long-term BTH2 treatment in cytokines levels of phytohemagglutinin-stimulated splenocytes. Figures S1–S6 used the same statistical analysis. One-way ANOVA with post-test of Tukey was used to verify statistical differences between the three mice groups. #Comparison with control. *Comparisons with Sham. *, # *p* ≤ 0.05; **, ## *p* <  0.01; ***, ### *p* < 0.001. ****, #### *p* <  0.0001. Figure S7: Correlation matrix between ratio of cytokine measured in the bronchoalveolar lavage fluid and cellular parameters. Figure S8: Correlation matrix between ratio of cytokine measured in lung homogenates and cellular parameters. Figure S9: Correlation matrix between ratio of cytokine measured in the supernatant of unstimulated splenocytes and cellular parameters. Figure S10: Correlation matrix between ratio of cytokine measured in the bronchoalveolar lavage fluid and antibodies measurements. Figure S11: Correlation matrix between ratio of cytokine measured in lung homogenates and antibodies measurements. Figure S12: Correlation matrix between ratio of cytokine measured in the supernatant of unstimulated splenocytes and antibodies measurements. For Figures S7–S12: Pearson or Spearman correlation coefficients were applied depending on data distribution. Color intensity and circle size represent the strength and direction of correlations. TCC, total cells count; EOS, eosinophils; NEU, neutrophils; MO, macrophages; LYM, lymphocytes; SI, stimulation index; EPO, eosinophil peroxidase, with letters “b” and “l” for BALF and lungs samples, respectively; for IgA antibodies, letters “s”, “b” and “l” mean IgA antibodies measured in sera, BALF and lungs, respectively; blo, blocking antibodies.
