# Supplementary figures and images for: Long-Term Administration of BTH2 Hypoallergenic Vaccine Candidate Induces Hallmarks of Allergen Immunotherapy in Murine Model of Blomia tropicalis-Induced Asthma
Source: Biomedicines. 2025 Oct 29;13(11):2657. doi: 10.3390/biomedicines13112657 (PMC12650233; doi:10.3390/biomedicines13112657)

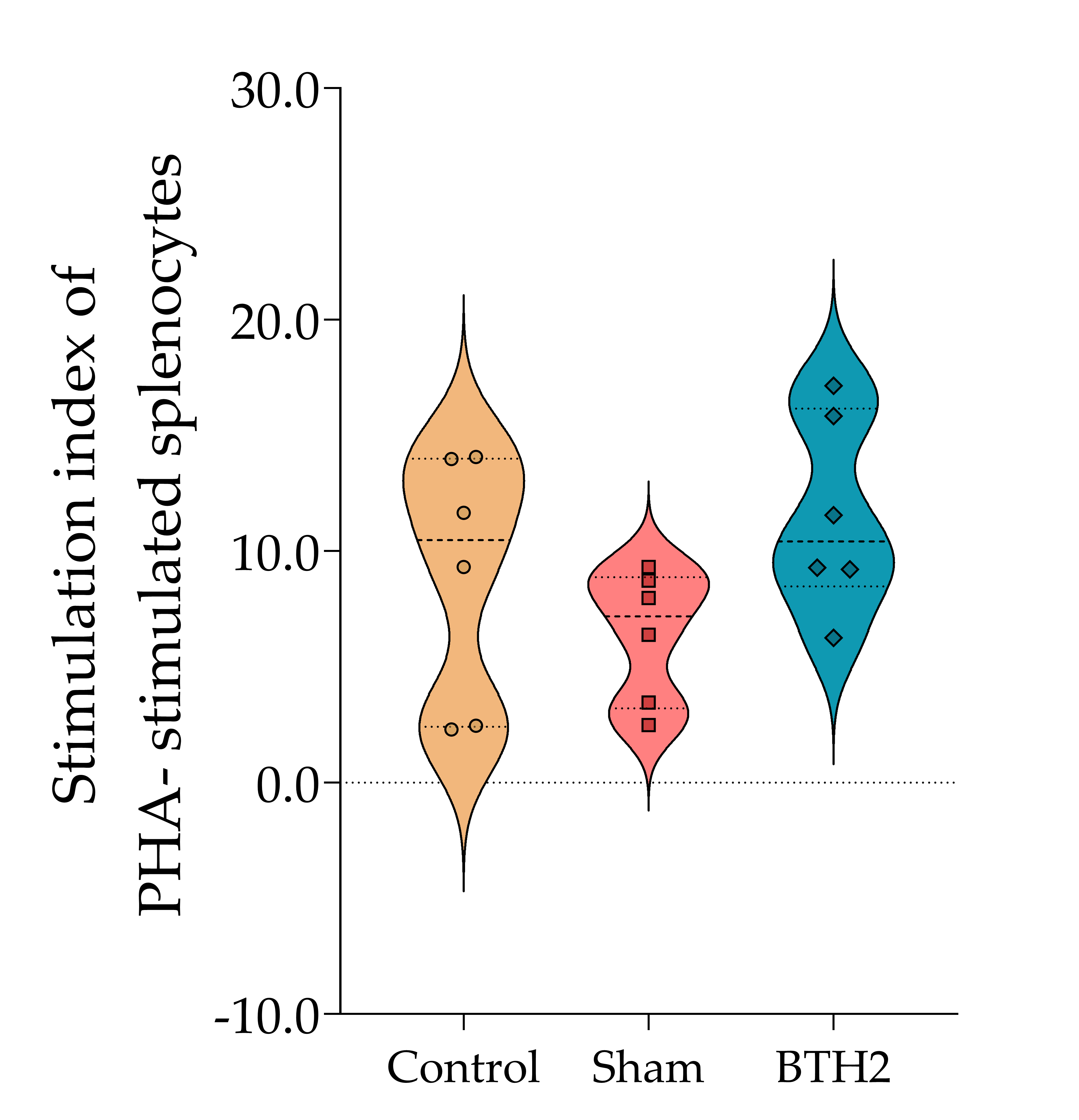

Supplement: Supplementary file 1 [file biomedicines-13-02657-s001.zip › supplementary_figures_biomedicines/Figure S1.png]

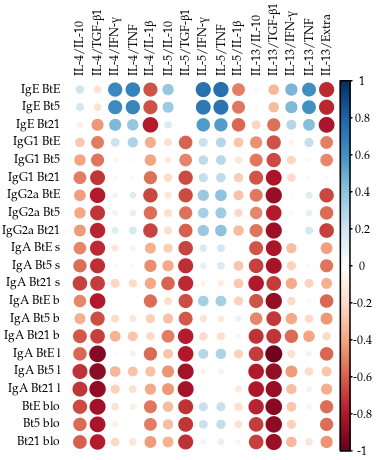

Supplement: Supplementary file 1 [file biomedicines-13-02657-s001.zip › supplementary_figures_biomedicines/Figure S10.png]

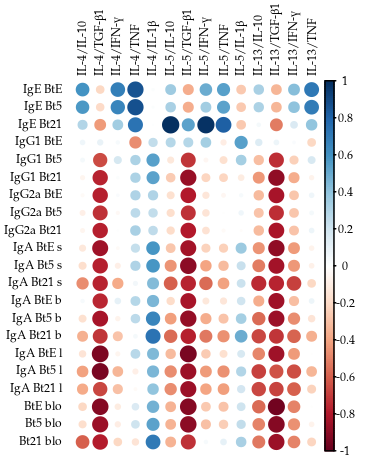

Supplement: Supplementary file 1 [file biomedicines-13-02657-s001.zip › supplementary_figures_biomedicines/Figure S11.png]

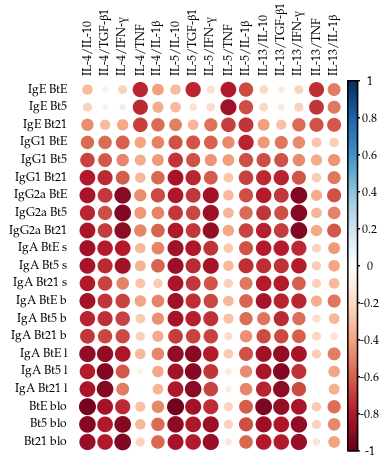

Supplement: Supplementary file 1 [file biomedicines-13-02657-s001.zip › supplementary_figures_biomedicines/Figure S12.png]

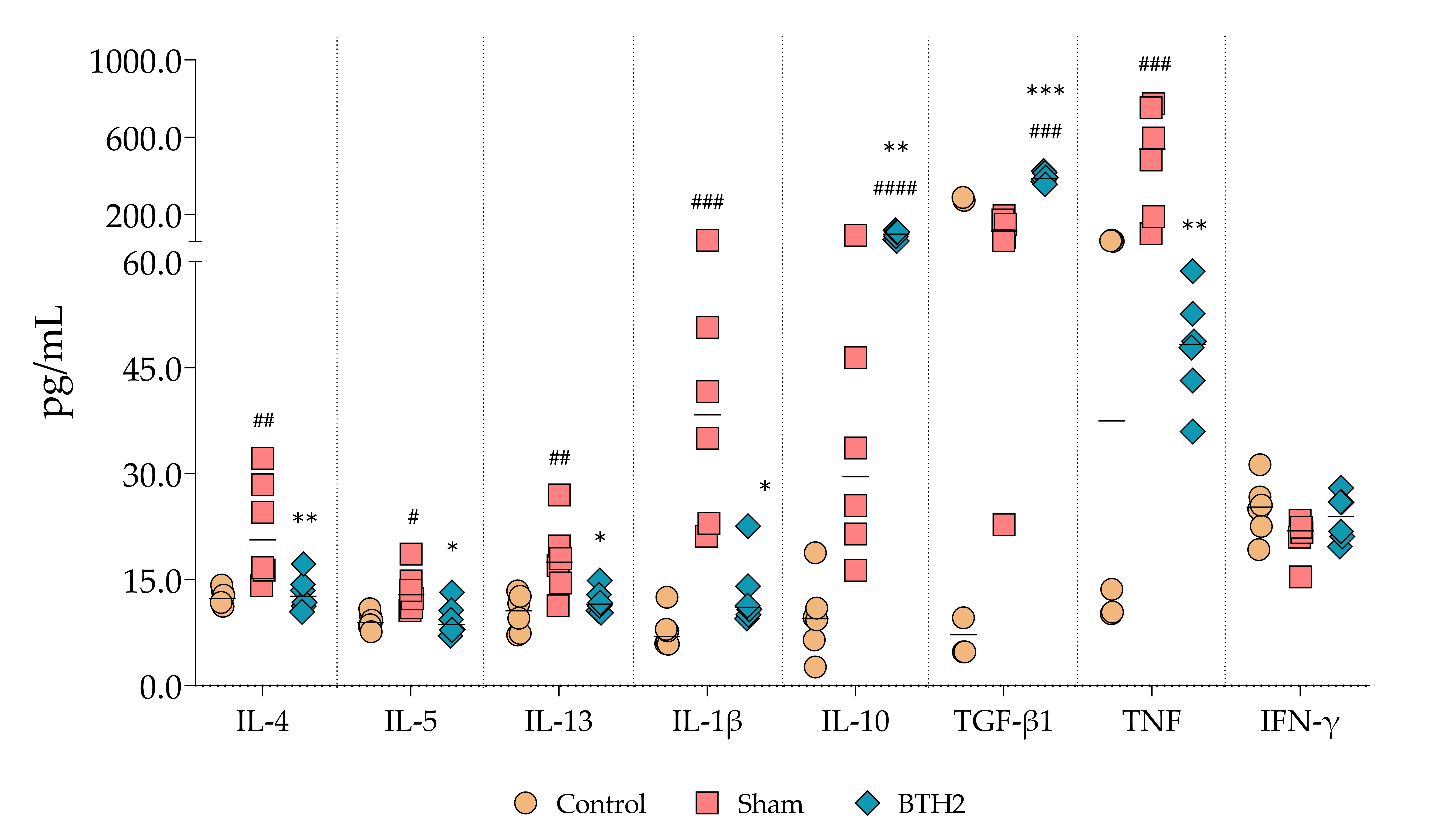

Supplement: Supplementary file 1 [file biomedicines-13-02657-s001.zip › supplementary_figures_biomedicines/Figure S2.png]

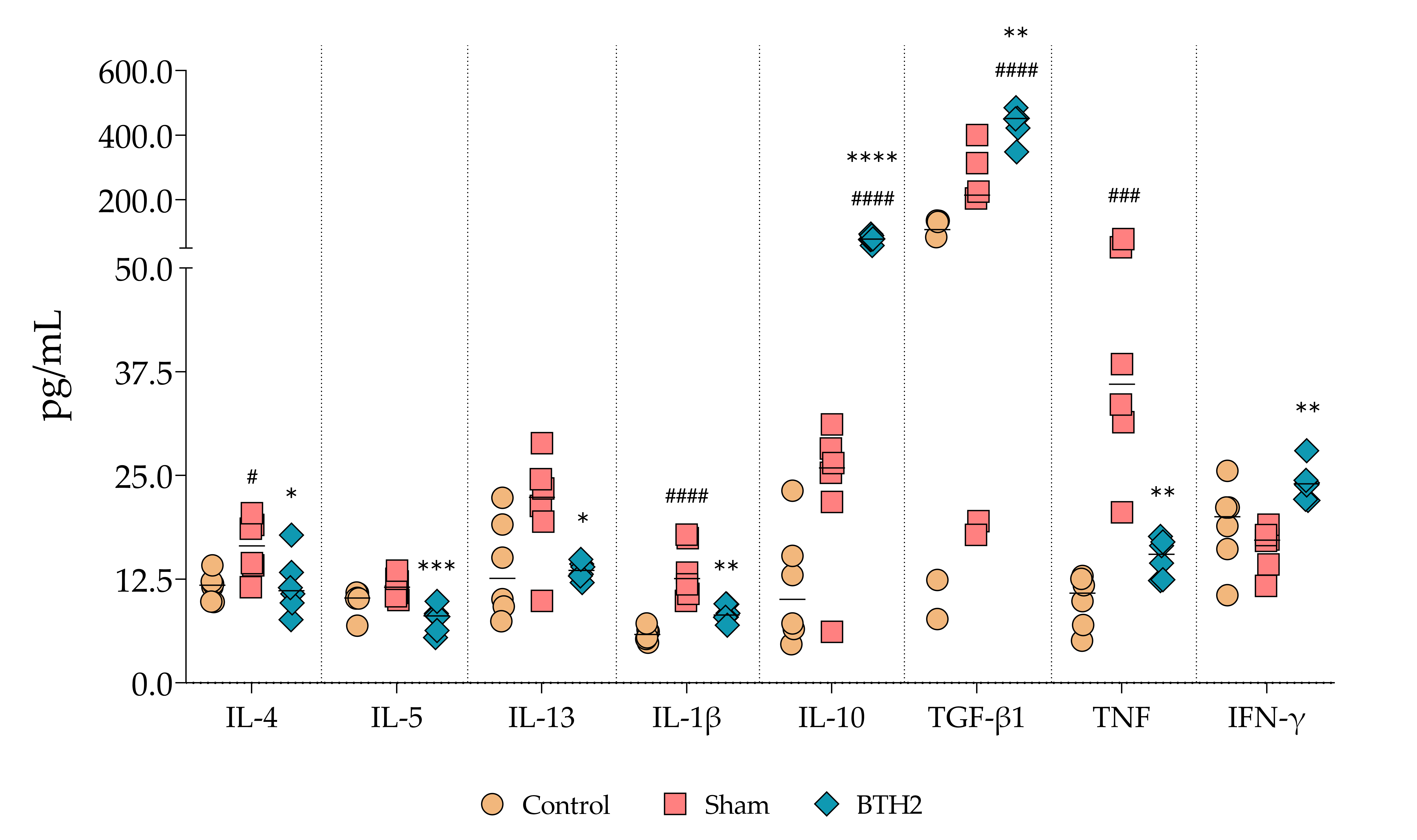

Supplement: Supplementary file 1 [file biomedicines-13-02657-s001.zip › supplementary_figures_biomedicines/Figure S3.png]

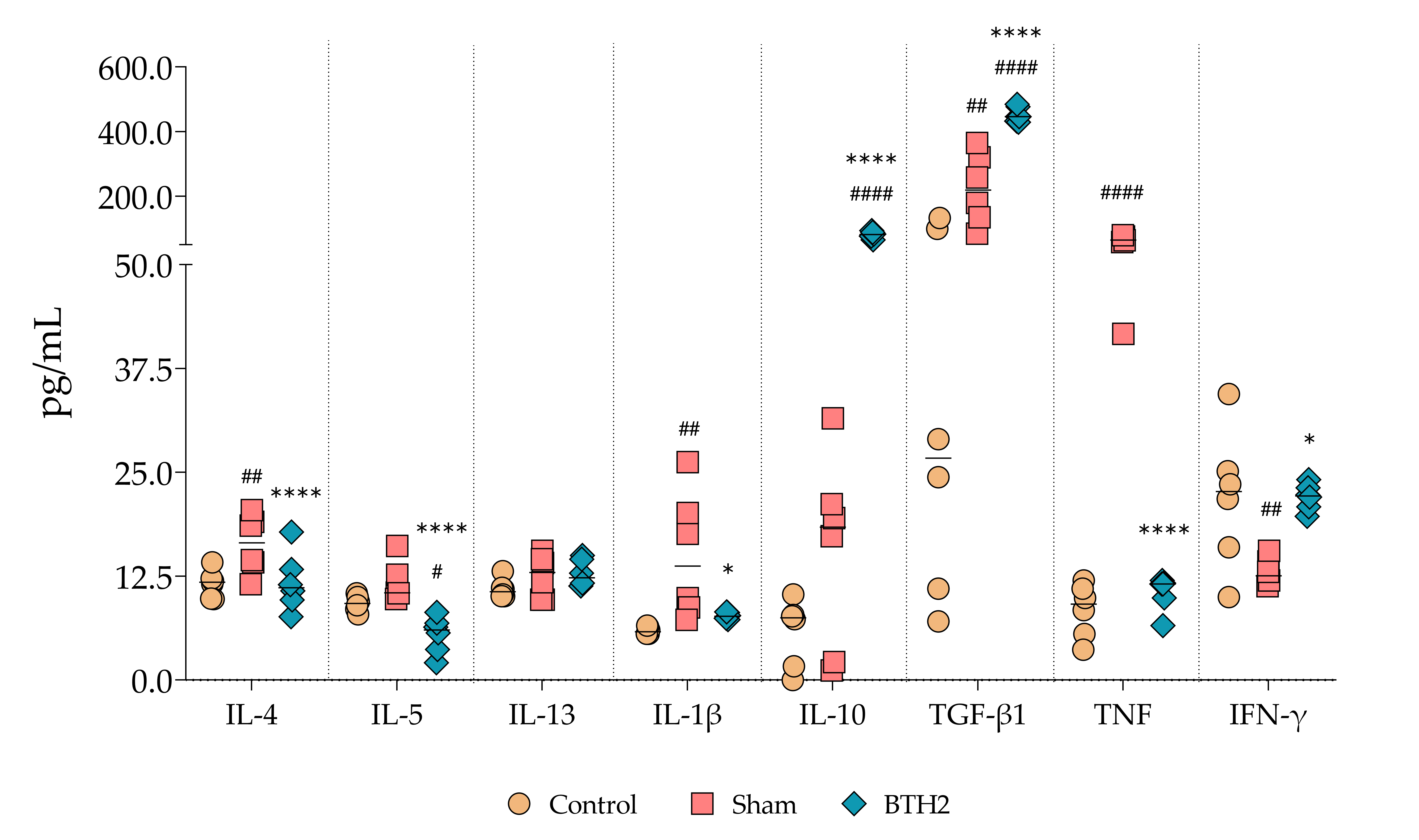

Supplement: Supplementary file 1 [file biomedicines-13-02657-s001.zip › supplementary_figures_biomedicines/Figure S4.png]

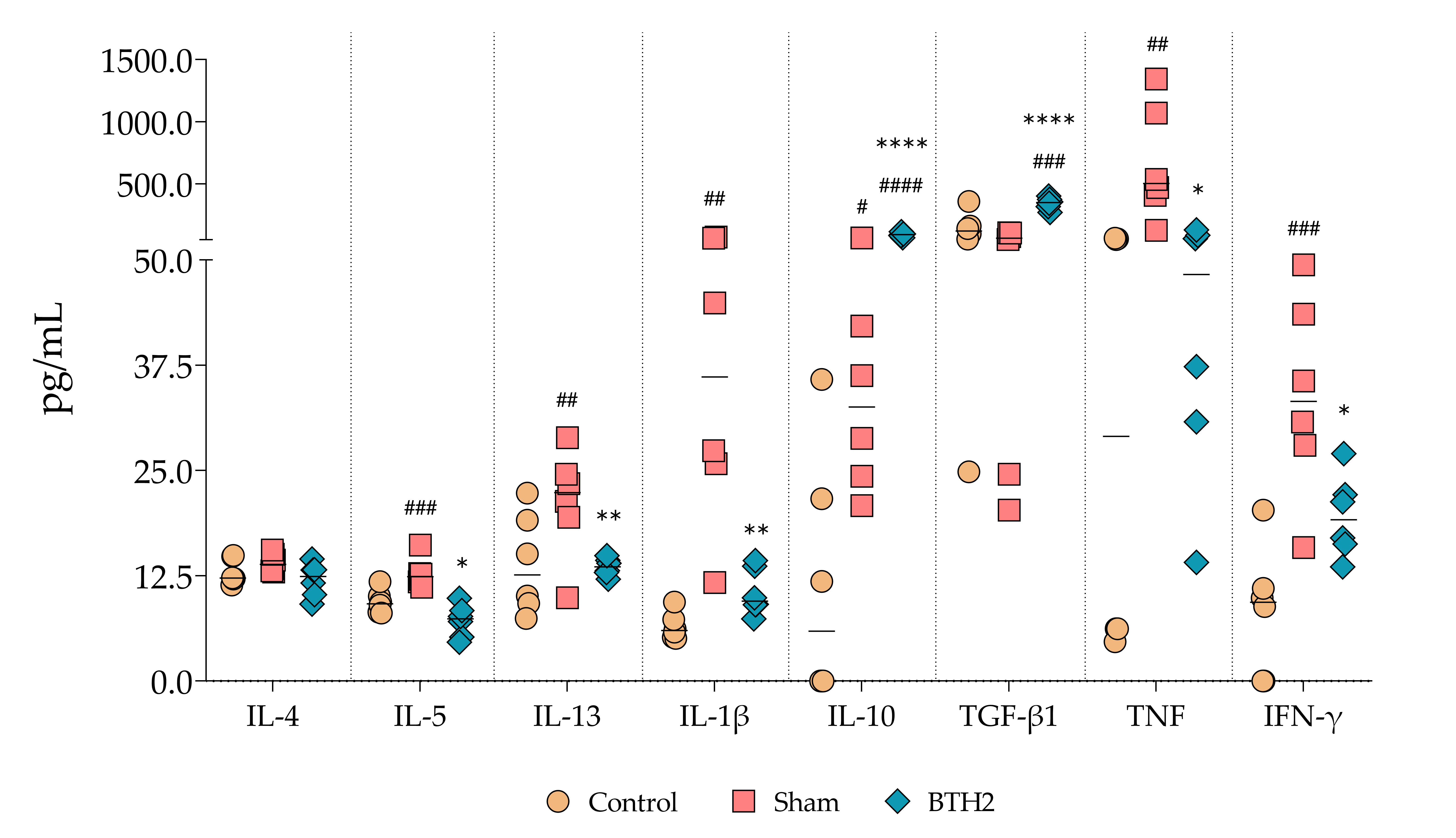

Supplement: Supplementary file 1 [file biomedicines-13-02657-s001.zip › supplementary_figures_biomedicines/Figure S5.png]

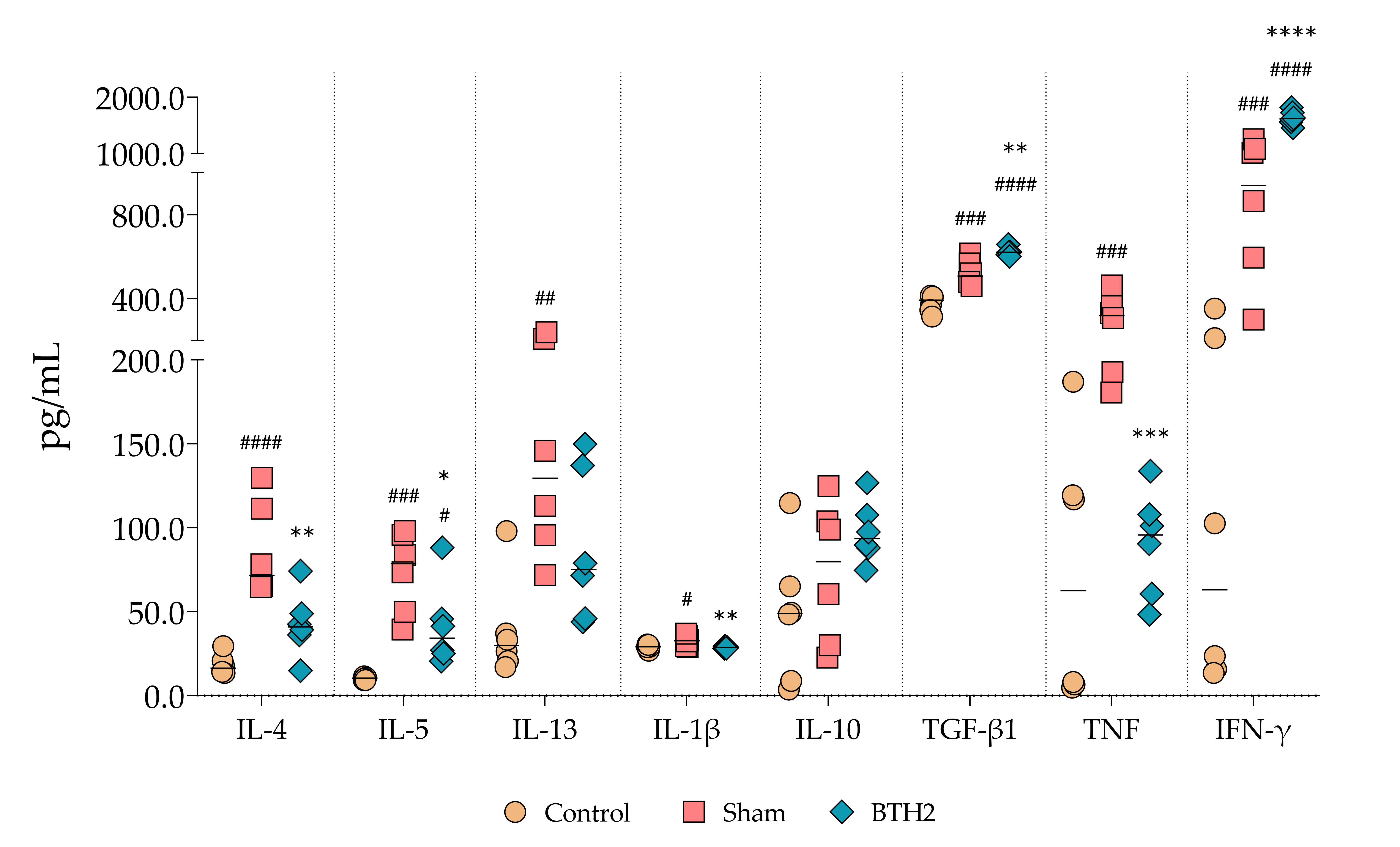

Supplement: Supplementary file 1 [file biomedicines-13-02657-s001.zip › supplementary_figures_biomedicines/Figure S6.png]

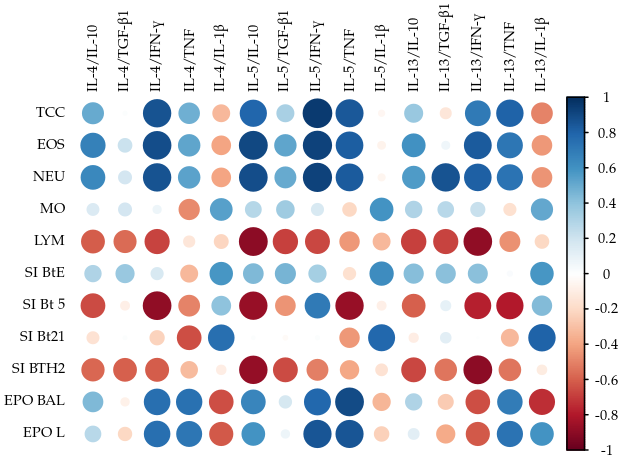

Supplement: Supplementary file 1 [file biomedicines-13-02657-s001.zip › supplementary_figures_biomedicines/Figure S7.png]

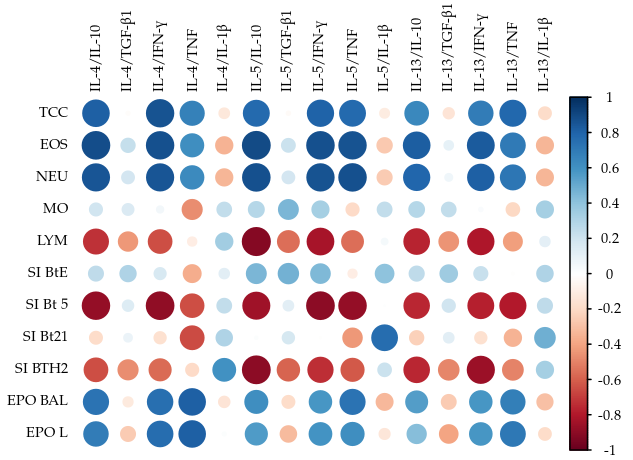

Supplement: Supplementary file 1 [file biomedicines-13-02657-s001.zip › supplementary_figures_biomedicines/Figure S8.png]

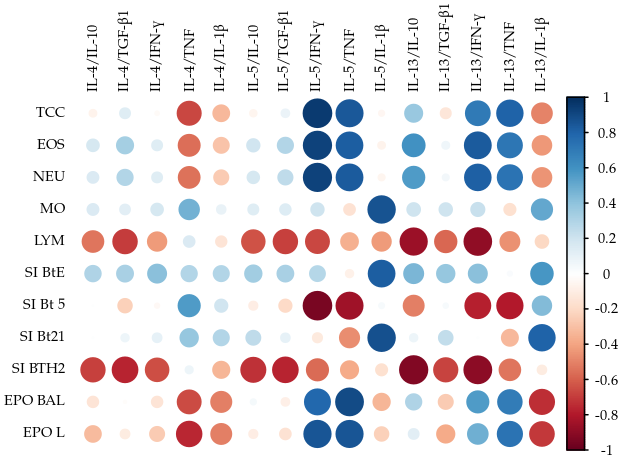

Supplement: Supplementary file 1 [file biomedicines-13-02657-s001.zip › supplementary_figures_biomedicines/Figure S9.png]
